# Interindividual Variability in Mental Fatigue-Related Impairments in Endurance Performance: A Systematic Review and Multiple Meta-regression

**DOI:** 10.1186/s40798-023-00559-7

**Published:** 2023-02-20

**Authors:** Jelle Habay, Robin Uylenbroeck, Ruben Van Droogenbroeck, Jonas De Wachter, Matthias Proost, Bruno Tassignon, Kevin De Pauw, Romain Meeusen, Nathalie Pattyn, Jeroen Van Cutsem, Bart Roelands

**Affiliations:** 1 Human Physiology and Sports Physiotherapy Research Group, Faculty of Physical Education and Physiotherapy, Vrije Universiteit Brussel, Pleinlaan 2, 1050 Brussels, Belgium; 2 BruBotics, Vrije Universiteit Brussel, Brussels, Belgium; 3 Vital Signs and Performance Monitoring Research Unit, LIFE Department, Royal Military Academy, Brussels, Belgium; 4 Research Foundation Flanders (FWO), Brussels, Belgium

**Keywords:** Mental fatigue, Physical performance, Individual response, Individual features, State, Trait, Systematic review, Meta-analysis, Meta-regression

## Abstract

**Background:**

The negative effect of mental fatigue (MF) on physical performance has recently been questioned. One reason behind this could lie in the interindividual differences in MF-susceptibility and the individual features influencing them. However, the range of individual differences in mental fatigue-susceptibility is not known, and there is no clear consensus on which individual features could be responsible for these differences.

**Objective:**

To give an overview of interindividual differences in the effects of MF on whole-body endurance performance, and individual features influencing this effect.

**Methods:**

The review was registered on the PROSPERO database (CRD42022293242). PubMed, Web of Science, SPORTDiscus and PsycINFO were searched until the 16th of June 2022 for studies detailing the effect of MF on dynamic maximal whole-body endurance performance. Studies needed to include healthy participants, describe at least one individual feature in participant characteristics, and apply at least one manipulation check. The Cochrane crossover risk of bias tool was used to assess risk of bias. The meta-analysis and regression were conducted in R.

**Results:**

Twenty-eight studies were included, with 23 added to the meta-analysis. Overall risk of bias of the included studies was high, with only three presenting an unclear or low rating. The meta-analysis shows the effect of MF on endurance performance was on average slightly negative (*g* = − 0.32, [95% CI − 0.46; − 0.18], *p* < 0.001). The multiple meta-regression showed no significant influences of the included features (i.e. age, sex, body mass index and physical fitness level) on MF-susceptibility.

**Conclusions:**

The present review confirmed the negative impact of MF on endurance performance. However, no individual features influencing MF-susceptibility were identified. This can partially be explained by the multiple methodological limitations such as underreporting of participant characteristics, lack of standardization across studies, and the restricted inclusion of potentially relevant variables. Future research should include a rigorous description of multiple different individual features (e.g., performance level, diet, etc.) to further elucidate MF mechanisms.

**Supplementary Information:**

The online version contains supplementary material available at 10.1186/s40798-023-00559-7.

## Key Points

- There is a large range of interindividual differences in the negative impact of mental fatigue on dynamic maximal whole-body endurance performance.
- No influence, both combined as well as isolated, on mental fatigue-susceptibility was found for the included individual features (i.e., age, sex, BMI and performance level).
- Further research is needed to understand the individual response to mental fatigue, and individual features possibly influencing this response.

## Introduction

Mental fatigue (MF) can be defined as a psychobiological state that arises during prolonged demanding cognitive, physical and/or emotional activity and results in an acute feeling of tiredness and/or a decreased performance capacity [1]. Throughout the years, multiple different terms for this complex phenomenon have been put forward (e.g., ego depletion [2], cognitive fatigue [3]) due to the isolated study of the MF concept in different research fields [4]. While there is merit in debating the exact nature and meaning of these terms (see Forestier et al. [5]), this review is not aimed at getting involved in this debate, and will, for the sake of clarity, be uniformly referring to this phenomenon as MF. Multiple studies have shown that MF causes decrements in specific forms of both cognitive (e.g. attention [6] and executive control [7]) and physical (e.g. endurance [8, 9] and sport-specific psychomotor [1]) performance. Meanwhile, numerous theories about the mechanisms underlying MF-effects have already been proposed across different research fields [2, 10–14]. However, the abundance of mechanisms remain largely theoretical, as current research is only able to provide indirect evidence [10, 11]. The true mechanisms of MF are therefore still to be elucidated, and many important aspects remain, at this point, underexamined [15].

More recently, the true effects of MF have been questioned by a bias sensitive meta-analysis, which concluded that the effect of MF is small and possibly insignificant [16]. Also, some multicentre replication studies examining MF-effects have failed to replicate them [17, 18]. This result is diametrically opposed to the findings of other meta-analyses [9, 14]. A potential explanation for these conflicting results might be the variability in individual responses to MF [19, 20]. Yet, this specific research-focus (i.e., assessing individual responses in MF-effects) has mostly been neglected. Ackerman et al. [21] already proposed in 2011 that interindividual differences are the most overlooked subject in the study of MF. Multiple, more recent, studies have agreed with this statement, and have mentioned that these differences are something important to keep in mind in future research [16, 19, 20, 22]. Noé et al. [19] noted a distinct amount of variability between individuals which allowed the authors to cluster participants based on their MF-susceptibility (i.e., the amount of MF a person suffers from and the influence it has on that person’s performance). To further explicate MF effects and its mechanisms, it is imperative that the individual responses to MF are taken into account and the possible reasons behind these interindividual differences are further examined [20]. For instance, further research could aim at uncovering the true nature of MF-susceptibility, being either state- (i.e., interindividual differences fluctuate in time) or trait (i.e., interindividual differences are robust in time) related [23]. MF in diseased populations (e.g., traumatic brain injury [24]) is seen as a trait [23], while it is generally accepted that acute MF induced in healthy individuals is a transient cognitive state (see definition provided [1]). However, while some preliminary research on this topic has already been carried out [23, 25], no clear consensus can yet be given on the nature of MF-susceptibility. Identifying this fundamental aspect of MF-susceptibility will be one of the ways that investigating interindividual differences will greatly enhance our understanding of MF, and the ways we investigate it.

One important way to evaluate the individual differences in MF-susceptibility is by investigating the role of individual features. This hypothesis is based on the established finding that some individual features (e.g. age [26], genetics [27], resilience [28]) affect cognitive performance and functioning. Hence, it could be argued that the same individual features influencing cognitive prowess might impact MF-associated decrements in physical performance. Some studies have already investigated the influence of individual features on MF-susceptibility. For example, Noé et al. [19] linked the variability in the level of MF and MF-susceptibility to associations between subjective responses, behavioural impairments and balance control. Moreover, other recent studies indicated that the physical fitness level of participants influences MF-susceptibility [29–34]. Other features investigated are age [35], sex [36], and self-regulation [37]. It should be mentioned that factors of a biological origin (e.g., age, fitness level) have been more extensively investigated compared to psychological factors (e.g., mental toughness [38], hardiness [39]). However, the exact role of each of these individual features in defining MF-susceptibility is yet to be accurately determined, given that no large study has examined these features in relation with one another. This limits researchers in their search for the underlying mechanisms of the impact of MF on physical performance, as well as practitioners who might use these features to identify and protect individuals who are more susceptible to MF. Moreover, an investigation into both biological and psychological features enables researchers to view MF-susceptibility in a truly holistic way, presenting findings simultaneously within two research domains that have already prominently, but largely independently, investigated MF and its effects on performance [4].

Therefore, the aim of the present systematic review and meta-analysis is not only to provide a potential range of interindividual differences in MF-effects, but also to identify, analyse and quantify individual features that might underlie these differences. Even though most MF reviews and meta-analyses have already incorporated various subgroup analyses, these subgroups are often based on differences in study methods and not on participant characteristics [9, 16, 40]. A multiple meta regression is more flexible and allows us to investigate different group specific effects and interaction effects with group indicators or other characteristics. A multiple meta-regression was thus performed to investigate the interaction effects of multiple individual features on MF-susceptibility.

## Methods

The present systematic review and meta-analysis was conducted in accordance with the updated “Preferred Reporting Items for Systematic review and Meta-analyses” (PRISMA) guidelines of 2020 [41]. The guidance for implementing PRISMA in exercise, rehabilitation, sport medicine and sports science (PERSiST) was also consulted [42]. The protocol was registered with PROSPERO: CRD42022293242.

### Eligibility Criteria

Studies were eligible when the studied population only included healthy individuals; when the target of the interventions was to induce MF; and when the study outcomes featured whole-body dynamic maximal endurance performance. The following terms were accepted as possible equivalents of MF: mental fatigue, cognitive fatigue, self-control strength depletion and ego depletion. All forms of MF interventions were accepted, as long as they consisted of a purely cognitive challenge (no dual tasks [8]) and the presence of MF was confirmed using some sort of manipulation check (s) (subjective, behavioural and/or physiological) [1]. In order for manipulation checks to be valid they clearly had to examine the degree of MF in participants. If not, the study was excluded. When available in studies, control tasks had to serve the purpose of not inducing MF or at least triggering less MF than the intervention task. Moreover, in studies without control tasks, the presence of a baseline measure providing a comparison in primary performance outcome between a mentally fatigued and a non-mentally fatigued state was mandatory. The physical performance outcome needed to be evaluated after the MF-inducing intervention. We chose to only include dynamic whole-body endurance performance as a physical outcome measure to keep the heterogeneity of the review and meta-analysis, that can be linked to both methodology and different theorised MF-mechanisms based on performance outcome, to a minimum. This type of physical performance has been consistently shown to be impacted by mental fatigue and is one of the most investigated aspects of physical performance within MF research [8, 9]. We defined dynamic whole-body endurance performance as: “performance during dynamic (i.e., in motion), whole-body (i.e., multiple different large muscle groups) exercise that involves continuous effort and lasts 75 s. or longer” [8, 43, 44]. Moreover, only tasks where participants were instructed to perform at their personal best were included. All experimental cross-over study designs (randomised controlled trials, non-randomised controlled trials or non-randomised non-controlled trials) which were published in peer-reviewed scientific journals were considered eligible for inclusion. Between-subjects study designs were excluded as it would be impossible to connect any individual features to MF effects. Lastly, all MF studies utilising additional interventions were also considered and included when the isolated effects of MF could be interpreted.

### Information Sources and Search Strategy

PubMed (MEDLINE) (Ovid) (sorted on best match), Web of Science (Core collection), PsycINFO (Ovid) and SPORTDiscus (EBSCOhost) were searched till the 16th of June 2022. There were no limits applied to the employed databases. All search strategies included, among others, combinations of the following terms: “mental fatigue”, “central fatigue”, “cognitive fatigue”, “cognitive exertion”, “mental exertion”, “mental strain”, “cognitive strain”, “ego depletion”, “performance”, “skills”, “speed”, “accuracy”, “physical”, “endurance”, “exercise”, “sport”, “psychomotor”, “neuromuscular”, “muscle”, and “isometric” (see also Table 1). Where possible, Medical Subject Headings (MeSH) terms were added to the search string. A backwards and forwards citation search of the included studies was also conducted using the Web of Science citation database. Lastly, to make sure no eligible studies were missed, the included article list of different topic-related systematic reviews [1, 8, 9, 14, 16, 45–52] were also examined, and potential eligible articles from these lists were included in the final study list of the present review.

**Table 1:** Search strategies and number of hits for the PubMed (MEDLINE), Web of Science, PsycINFO and SPORTDiscus databases

Database	Interface/platform	Complete search strategy	Hits (16/06/2022)
PubMed	Ovid	((((((((("mental fatigue") OR ("central fatigue")) OR ("cognitive fatigue")) OR ("cognitive exertion")) OR ("mental exertion")) OR ("mental strain")) OR ("cognitive strain")) OR ("ego depletion")) OR ("mental fatigue"[MeSH Terms])) AND ((((((((((((((performance) OR (skills)) OR (speed)) OR (accuracy)) OR (physical)) OR (endurance)) OR (exercise)) OR (sport)) OR (psychomotor)) OR (neuromuscular)) OR (muscle)) OR (isometric)) OR ("psychomotor performance"[MeSH Terms])) OR ("athletic performance"[MeSH Terms]))	3400
Web of Science	/	TS = ((performance OR skills OR speed OR accuracy OR physical OR endurance OR exercise OR sport OR psychomotor OR neuromuscular OR muscle OR isometric) AND ("mental strain" OR "cognitive strain" OR "mental fatigue" OR "central fatigue" OR "cognitive fatigue" OR "cognitive exertion" OR "mental exertion" OR "ego depletion" OR “self-control strength depletion”))	4240
PsycINFO	Ovid	(performance OR skills OR speed OR accuracy OR physical OR endurance OR exercise OR sport OR psychomotor OR neuromuscular OR muscle OR isometric) AND ("mental fatigue" OR "central fatigue" OR "cognitive fatigue" OR "central fatigue" OR "cognitive exertion" OR "mental exertion" OR "mental strain" OR "cognitive strain" OR "self-control strength depletion" OR "ego depletion")	1681
SPORTDiscus	EBSCOhost	( performance OR skills OR speed OR accuracy OR physical OR endurance OR exercise OR sport OR psychomotor OR neuromuscular OR muscle OR isometric) AND ( "mental fatigue" OR "central fatigue" OR "cognitive fatigue" OR "cognitive exertion" OR "mental exertion" OR "mental strain" OR "self-control strength depletion" OR "ego depletion")	1020

### Study Selection Process

Articles were gathered from all databases, while duplicates were removed using Endnote version X9.3.3. Afterwards, this pool of studies was imported into Rayyan [40], where two authors (J.H. and R.U.) independently screened all articles on title and abstract for eligibility. In a subsequent meeting the two authors resolved any conflicts arising from the first screening stage. The second screening stage featured the same two authors independently screening the full texts and performing a last meeting to resolve full text inclusion conflicts. The number of conflicts related to the total number of articles was 230 (3.5%) in the first stage and 3 (1.2%) in the second stage. Any conflicts arising from the proposed meetings between the two authors were resolved through consensus or through a general meeting with all other authors.

### Data Collection Process, Items and Categorization

The effects of MF on endurance performance were collected from the included articles. One author (J.H.) extracted relevant data from the included articles, the correctness of which was checked by a second author (M.P.). Discrepancies between authors were resolved through discussion. Critical data that needed to be collected included the used physical task, any individual characteristics of the participants, and the effect of the intervention with suitable effect size. Other important information included the study design, intervention details, manipulation checks, sample size, treatment groups, control and statistical analysis. No changes were made to the inclusion/definition of the proposed primary and secondary outcomes or to the importance given to them throughout the data collection process of the present review. In case of missing data in included studies, corresponding authors were contacted to either provide the data or a reason for omittance. If no answer was received, the missing data were not further pursued and were, if relevant, added to the risk of bias assessment. Overall, about 38% of included studies did not describe all included participant characteristics, which changed to 35% after contacting the different authors, and decreased to 16% after calculation of variables (concerning the studies that were eventually included in the meta-analysis, and keeping in mind the variables that were chosen for the meta regression analysis).

The following individual features (and the reason behind their selection) were analysed using a multiple meta-regression: age (chosen based on deterioration of cognitive abilities with old age and the development of the brain during youth [53, 54]), biological sex (chosen based on the different ways that men and women cope with mental load [36, 55]), anthropometric measures (based on a link between body mass index (BMI)/body fat percentage, a typical measure of health, and cognitive abilities [56]) and training level (based on the hypothesized cognitive abilities of elite athletes [31, 57] and the possible way to train MF-susceptibility with endurance training [32]). These features encapsulate the following outcomes (both given by the studies as well as calculated by the author team): mean age and age categories, number of men, number of women and sex distribution (number of women/total sample size), mean mass, height, body fat percentage and BMI, physical fitness level [58, 59], peak power output (PPO), VO_2_peak/max, mean years of experience and category of years of experience, mean training frequency (sessions/week) and category of training frequency, mean training load (km/week) and category of training load, and mean training volume (hrs/week) and category of training volume. Participants were categorized by physical fitness level based on the performance levels proposed by both De Pauw et al. [58] (men) and Decroix et al. [59] (women). In groups utilising both men and women, performance levels were categorised based on the sex that was most prevalent (n studies = 5). If no relevant performance level metrics were present, the VO_2_max was calculated using the results of the Yo-Yo intermittent recovery (IR) test [60] and the beep test [61] using the appropriate formulas (if these metrics were present in the included study), which was then connected to the appropriate performance level. The above-mentioned factors were selected based on the most prominently described participant characteristics of the eventually included articles.

### Risk of Bias Assessment

Since only within-subjects study designs were eventually included in the present review, the Revised Cochrane Risk of Bias (RoB) tool for cross-over trials as proposed by Ding et al. [62] was used to determine risk of bias of the included studies. The RoB screening was done independently by two authors (J.D.W. and R.U.). Based on the signalling questions provided in the RoB tool, each of the proposed domains received a rating which was either “low risk of bias”, “high risk of bias” or “some concerns of bias”. Finally, an overall risk of bias judgement was made for each study (i.e., “low”, “high” or “unclear”). The authors followed the guidelines provided by the Cochrane community. Disagreements between authors were resolved through discussion. If no consensus could be reached, a third author (J.H.) was consulted who reached a final verdict based on the comments of both authors and an independent screening of the articles that were the subject of discussion. RoB results were visualised by the robvis tool (https://mcguinlu.shinyapps.io/robvis/).

### Synthesis Methods and Effect Measures

All studies were summarized in a comprehensive table featuring study name, available participant characteristics, intervention and control task, duration of tasks, manipulation checks and physical outcome measures. The meta-analysis and meta-regression analysis were performed using the meta (version 5.2-0) [63], metafor (version 3.4-0) [64], and dmetar (version 0.0.9000) [65] packages in R (version 4.1.2.). Based on previous systematic analyses [9, 16, 40] of the MF literature, a random-effects model was adopted with Hedges’ g used as the definitive effect size [66]. These effect sizes were calculated in Excel and checked using the dmetar package. If studies did not report the true means and standard deviations, the authors were contacted to provide this or the original data. 80% of authors contacted provided us with their data to be used in the eventual meta-analysis. If authors were unable to provide the data, but the data were depicted in figures, the means and standard deviations were extracted from the figures using GetData Graph Digitizer 2.26 software. Fatigue effects can be represented by both an increase or a decrease in outcome variables (e.g., the duration to overcome a specific distance in a time trial is suspected to increase when fatigued, while TTE is suspected to decrease when fatigued). Therefore, to improve readability, all MF effects in which an increase in the primary outcome is related to an impairment in performance were inverted, so that all negative/impairing effects also resulted in a negative effect size. If multiple physical performance outcome measures were available, the outcome that best represented dynamic maximal whole body endurance performance was selected for the meta-analysis. If studies incorporated different groups, based on outcome measure or population, these groups were seen as distinct effects, and within study similarities were ignored. Tau and tau squared values were calculated using the restricted maximum likelihood procedure. Between-study heterogeneity is displayed using Cochran’s Q (represents the weighted sum of squares) and I^2^ (representing the amount of variability in effect size outcomes that is not caused by the sampling error). High, moderate and low amounts of heterogeneity are represented by an I^2^ value of 75%, 50%, and 25% respectively. Publication bias was assessed visually using Funnel plots and quantitatively using Egger’s regression test and Rosenthal’s fail safe N. Forest plots were also generated with the forest.meta function in R. A meta-regression was performed in order to examine the influence of internal factors on the effects of MF on endurance performance. Multi-collinearity was first checked for the eventual included factors, using both quantitative data and figures. Afterwards, the rma function in R was used to perform the multiple linear meta-regression. Knapp-Hartung adjustments were used to reduce the risk of false significant effects. Estimates were removed one by one based on the estimate to attempt at improving the meta-regression model. The level of significance was set at *p* < 0.05.

All data (Excel file with effect size measures and study data, and R work files) are presented in Additional file 1 and Additional file 2.

## Results

### Study Selection

**Fig. 1:**
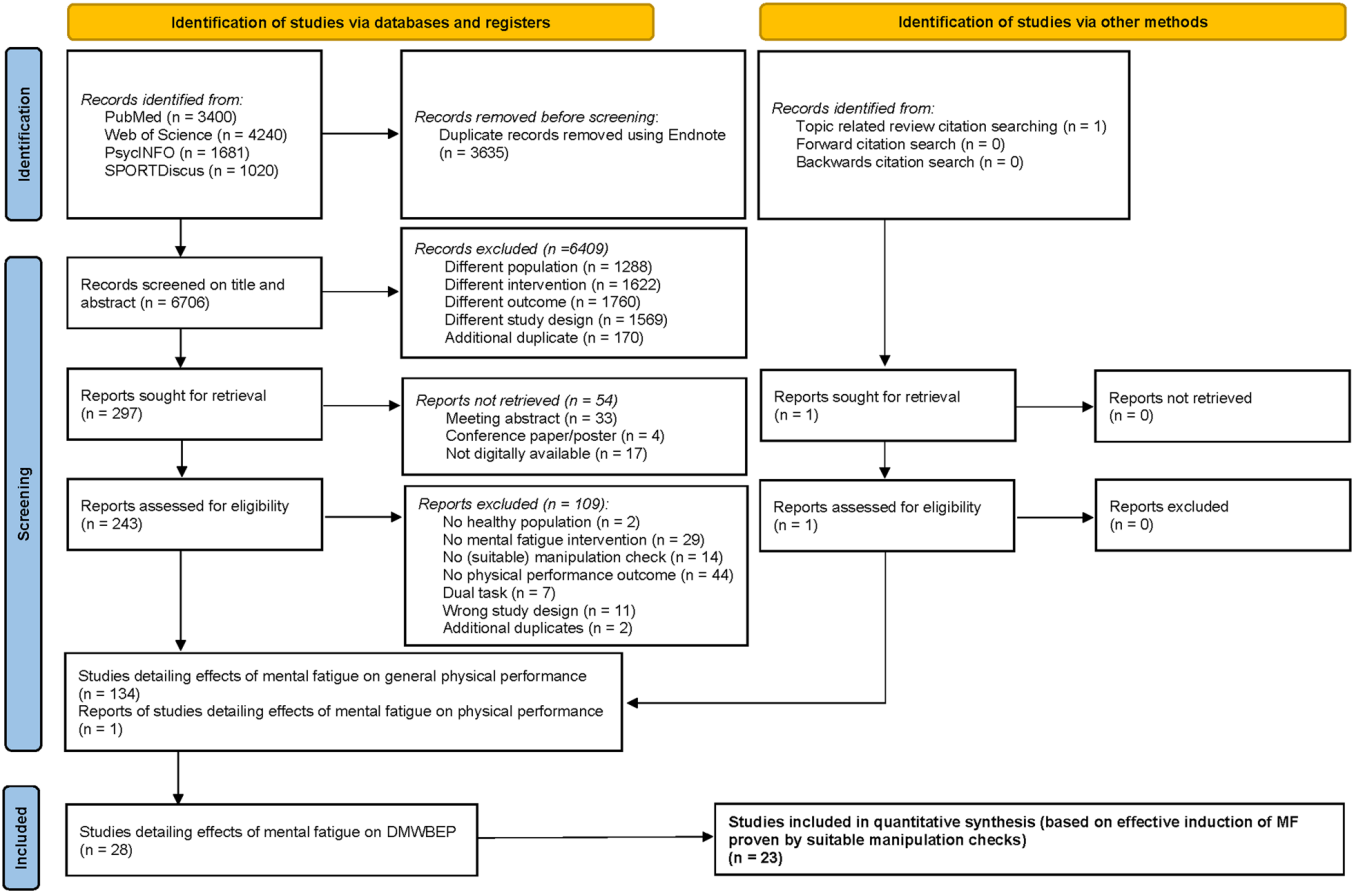
Prisma chart *DMWBEP* dynamic maximal whole body endurance performance

The original paper pool featured 10,341 articles, which was reduced to 6706 original and distinct articles after the removal of duplicates (see Prisma Chart, Fig. 1). 243 full texts were eventually read and subjected to the aforementioned inclusion criteria: healthy population, manipulation check presence, goal to induce MF and a physical outcome measure. Other exclusion reasons included the use of a dual physical and cognitive performance task as primary outcome, a non-suitable study design and additional duplicates not noticed in Endnote. This finally resulted in 134 articles detailing the effects of MF on physical performance, with 1 report added based on the reference list search. Eventually, 28 studies that detailed an investigation of MF-effects on dynamic maximal whole-body endurance performance were selected and included in the present review.

### Study Characteristics and Individual Features

**Fig. 2:**
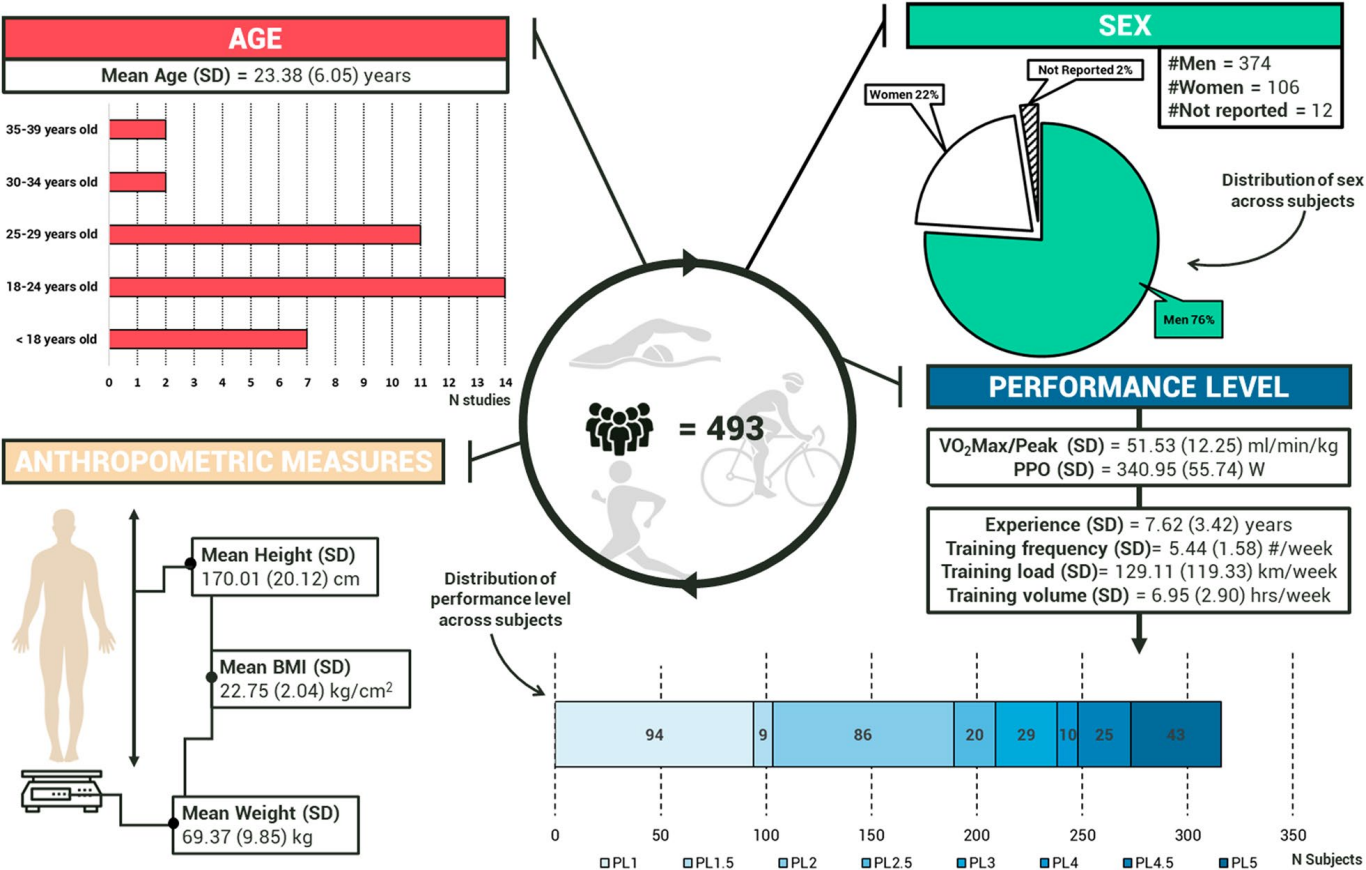
Schematic overview of the included individual features of the selected studies (*PL* performance level; Icons courtesy of the Noun Project.com (artists: Adrien Cocquet, Guilherme Silva Soares, Aleksandr Vector, Monkik))

All relevant information regarding study characteristics, MF tasks and manipulation checks, and effects on physical performance outcomes can be found in Table 2. Figure 2 depicts the reporting of different individual features of the included studies. Studies which included very distinct features only once (e.g., fat percentage [29], caffeine consumption [67]) were not included in this figure.

**Table 2:** Overview of the included studies, with participants characteristics, mental fatigue task aspects and overall effects on endurance performance. Effects on endurance performance attributed to mental fatigue are highlighted in bold

References	Participant characteristics	Intervention	Control	Duration	Manipulation check	Effect on performance (summary)
Brietzke et al. [79]	*N:* 20 cyclists (♂) *Age:* 35.00 ± 7.00 y *Mass:* 80.50 ± 10.40 kg *Height:* 176.00 ± 5.00 cm *BMI:* 26.04 ± 2.94 kg/m^2^ *Level:* PL 2/3 (PPO = 358.63 ± 21.96 W, YoE = 7.56 ± 5.89 y, TV = 310.6 ± 128.1 km/week)	RVIP test (PC)	–	40 min	*S:* MF ↑ in I_2_ vs I_1_ (assessed using M-VAS) *B:* ↓ in RT and ACC in I_2_ vs I_1_ (assessed during RVIP) *P:* ↑ θ band activity in I_2_ vs I_1_ (assessed using EEG)	**↓ in TTE (small) and PPO (small) in I vs Baseline, no effects on VO**_**2**_**Peak or VT (measured using a MIET) (ES(d))**
Campos et al. [29]	*N:* 13 judo athletes (9♂/4♀) *Age:* 19.50 ± 3.00 y *Mass:* 68.10 ± 13.30 kg *Height:* 169.00 ± 5.10 cm *Body fat:* 12.20 ± 3.80% *Level:* NA *(*YoE = 7.20 ± 3.90 y; TL = 11.20 ± 4.60 h/wk)	Incongruent Stroop task with RI (paper version)	Docu	30 min	*S:* MF ↑ in I_2_ vs I_1_ and in I_2_ vs C_2_ (assessed using M-VAS)	**No significant effects on number of throws (trivial) or physical performance index (small) (measured using a specific judo fitness test)**
Clark et al. [33]	(1) Competitive group: *N:* 10 competitive athletes (♂) *Age:* 27.40 ± 6.30 y *Mass:* 75.60 ± 9.70 kg *Height:* 179.00 ± 6.00 cm *Level:* PL3 (VO_2_ peak = 58.30 ± 4.10 mL/kg.min; PPO = 401.00 ± 36.00 W; W_get_ = 149.00 ± 32.00; TL = 9.50 ± 2.50 h/week; PA-R = 9.70 ± 0.70; TET = 9.50 ± 2.50 h/week) (2) Non-competitive group: *N:* 10 healthy participants (♂) *Age:* 25.80 ± 4.60 y *Mass:* 93.30 ± 17.00 kg *Height:* 183.00 ± 6.00 cm *Level:* PL1 (VO_2_ peak = 39.00 ± 7.30 ml/kg.min; PPO = 280.00 ± 27.00 W; W_get_ = 91.00 ± 25.00 W; TL = 3.20 ± 1.90 h/week; PA-R = 3.80 ± 1.90; TET = 3.20 ± 1.90 h/week)	Modified (congruent, incongruent and neutral) Stroop task and N-back task (PC)	Docu	30 min	*B:* No effects on ACC or RT in both groups (assessed during the intervention tasks) *P:* No effects on cerebral oxygenation in both groups (assessed using fNIRS)	**No evidence of MF based on manipulation checks**
Filipas et al. [68]	*N:* 18 rowers (11♂/6♀) *Age:* 11.00 ± 1.06 y *Mass:* 46.72 ± 11.14 kg *Height:* 154.79 ± 9.41 cm *Level:* NA (TF: > 2 sessions/week; YoE; 1.50 ± 0.85 y)	(1) Incongruent Stroop task (PC) (2) Customised arithmetic test (Paper)	Drawing a mandala	60 min	*B:* no effects on RT, ↑ in ACC in I_6_ vs I_1_ (assessed during the Stroop task)	**No evidence of MF based on manipulation checks**
Filipas et al. [69]	*N:* 10 U23 cyclists (♂) *Age:* 20.00 ± 1.20 y *Mass:* 66.10 ± 7.60 kg *Height:* 180.40 ± 5.60 cm *Level:* PL4 (calc.) (VO_2_Max = 69.00 ± 4.40 ml/min.kg; PPO = 380.00 ± 39.00 W; TF > 4 sessions/week; TL > 300 h/week; YoE > 3 y)	Modified (congruent and incongruent) Stroop task (PC)	Docu	30 min	*B:* no effects on RT and ACC (assessed during Stroop task) *P:* no effects on HRV (assessed using a Polar T61)	**No evidence of MF based on manipulation checks**
Filipas et al. [35]	(1) U14 group: *N:* 12 soccer players U14 (♂) *Mass:* 55.00 ± 8.00 kg *Height:* 168.00 ± 4.00 cm *Level:* NA (Exp = competing at national level; YoE > 3 y) (2) U16 group: *N:* 12 soccer players U16 (♂) *Mass:* 62.00 ± 8.00 kg *Height:* 170.00 ± 5.00 cm *Level:* NA (Exp = competing at national level; YoE > 3 y) (3) U18 group: *N:* 12 soccer players U18 (♂) *Mass:* 69.00 ± 8.00 kg *Height:* 177.00 ± 7.00 cm *Level:* NA (Exp = competing at national level; YoE > 3 y)	Incongruent Stroop task (PC)	Reading a selection of emotionally neutral online magazines	I: 30 min C: 15 min	*S:* MF ↑ in I vs C (assessed using M-VAS) *B:* No effects on ACC and RT (assessed during Stroop task)	**↓ in test distance (small to large) in I vs C in all age groups (measured using the Yo-Yo IR test) (ES(d))**
Filipas et al. [32]	(1) Training group: *N:* 10 participants (3♂/7♀) *Age:* 27.60 ± 6.30 y *Mass:* 69.60 ± 18.40 kg *Height:* 169.40 ± 6.80 cm *Level:* PL1 (calc.) (VO_2_peak = 32.90 ± 6.90 ml/min.kg) (2) Placebo group: *N:* 10 participants (3♂/7♀) *Age:* 27.50 ± 6.00 y *Mass:* 68.70 ± 14.30 kg *Height:* 169.50 ± 9.60 cm *Level:* PL1 (calc.) (VO_2_peak = 32.80 ± 5.60 ml/min.kg)	(1) Cognitive test battery (45 min) (2) Modified Stroop task (40 min) (3) task switching (5 min)	Staring at a white screen	I: 90 min C: 15 min	*S:* MF ↑ in I vs C (assessed using M-VAS)	**↓ in total distance (small) in I vs C in both groups (measured using a time-based time trial) (ES(d))**
Fortes et al. [87]	*N*: 25 swimmers (14♂/11♀) *Age:* 20.40 ± 2.06 y *Mass:* 72.00 ± 9.00 kg *Height:* 181.00 ± 7.00 cm *Level:* NA (TF = 5.80 ± 0.50 sessions/week; TL = 42.50 ± 6.20 km/week; YoE = ~ 8.40 y; FINApoints = 602.61 ± 25.78 (♂)/611.84 ± 26.61 (♀); Exp = international-level)	Smartphone use (Social networking apps)	Docu	30 min	*S:* MF ↑ in I vs C and in I_1_ vs I_2_ (assessed using M-VAS) *B:* no effect on ACC, ↑ in RT in I vs C (assessed using a short Stroop task) *P:* no effects on HRV (assessed using a Polar H10)	**↑ in completion time (medium) in I vs C (mostly visible in the second block) (assessed using a 200 m (distance based) swimming trial) (ES(η**^**2**^**))**
Franco- Alvarenga et al. [67]	*N:* 12 cyclists (?) *Age:* 34.30 ± 6.20 y *Mass:* 77.60 ± 6.80 kg *Height:* 179.30 ± 5.10 cm *Level:* PL3 (VO_2_Max = 58.90 ± 6.20 mL/kg.min; PPO = 367.00 ± 32.50 W; TF = 4.70 ± 2.30 sessions/wk; TL = 283.70 ± 138.60 km/wk; Experience = ~ 6.50 y) *Caffeine consumption:* non-consumers (≤ 40 mg/day) = 3; occasional consumers (≤ 250 mg/day) = 5; daily consumers (between 250 and 572 mg/day) = 4	RVIP test (PC)	–	40 min	*S:* MF ↑ in I_2_ vs I_1_ (assessed using M-VAS) *P:* ↑ in θ activity in I_2_ vs I_1_ (assessed using EEG)	**↑ in completion time (very large) and ↓ in APO (very large) in I vs C (assessed using a distance based time trial) (ES(d))**
Holgado et al. [77]	*N:* 30 participants (24♂/6♀) *Age:* 23.50 ± 6.30 y *Level:* PL1 (calc.) (VO_2_max: 41.88 ± 9.08 ml/min.kg)	AX-CPT (PC)	Documentary	90 min	*S:* MF ↑ in I vs C (assessed using M-VAS) *B:* no effects on ACC (assessed during the AX-CPT task)	**No effects on a TTE (assessed using a 80% VO**_**2**_** Max TTE test)**
Lam et al. [76] study 1	*N:* 9 participants (♂) *Age:* 22.00 ± 2.60 y *Mass:*70.00 ± 7.50 kg *Height:* 173.00 ± 2.00 cm	Incongruent Stroop task	/	30 min	*S:* MF ↑ in I vs Baseline (assessed using M-VAS)	**↓ in distance covered (small) in I vs Baseline (assessed using a Yo-Yo IR1 test) (ES(d))**
Lam et al. [76] study 2	*N:* 9 participants (7♂/2♀) *Age:* 21.10 ± 1.20 y *Mass:*74.00 ± 8.50 kg *Height:* 179.00 ± 4.00 cm	Incongruent Stroop task	–	30 min	*S:* MF ↑ in I vs Baseline (assessed using M-VAS)	**↑ in completion time (small) in I vs Baseline (assessed using a distance based time trial) (ES(d))**
Lopes et al. [36]	(1) Male group: *N:* 18 runners (♂) *Age:* 25.00 ± 1.00 y *Mass:* 63.20 ± 1.30 kg *Height:* 172.70 ± 1.50 cm Level: PL5 (calc.) (IAAF score = 846.00 ± 49.00; TF = 8 sessions/wk; TL = 12 ± 1 km/session or 96 ± 9 km/week; RE = 215.00 ± 4.00 ml/kg.km; VO_2_Max = 73.24 ± 1.37 ML/kg.min; maxHR = 191.00 ± 2.00; MaxSpeed = 21.90 ± 0.30 km/h; V_E_ = 160.00 ± 4.20 l/min) (2) Female group: *N:* 17 runners (♀) *Age:* 25.00 ± 1.00 y *Mass:* 52.40 ± 1.00 kg *Height:* 163.80 ± 1.20 cm Level: PL5 (calc.) (IAAF score = 867.00 ± 36.00; TF = 7 sessions/wk; TL = 12 ± 1 km/session or 88 ± 7 km/week; RE = 229.00 ± 4.00 ml/kg.km; VO_2_Max = 61.29 ± 1.45 mL/kg.min; maxHR = 186.00 ± 2.00; MaxSpeed = 18.80 ± 0.30 km/h; V_E_ = 112.00 ± 2.40 l/min)	Stroop task (PC)	Docu	45 min	*S:* MF ↑ in I_2_ vs C_2_ and in I_2_ vs I_1_ in both groups (assessed using M-VAS) *B:* no effects on RT and ACC (assess using a shorter and distinct Stroop task)	**↓ in TTE (small) in I vs C in both groups (measured using a running TTE test) (ES(d))**
Macmahon et al. [3]	*N:* 20 participants (18♂/2♀) *Age:* 25.40 ± 3.24 y *Level:* NA (TF = 2.84 ± 1.79 h/week)	AX-CPT	Docu	90 min	*B:* no difference in I vs C (assessed using AX-CPT)	**No evidence of MF based on manipulation checks**
Macmahon et al. [71]	*N:* 13 participants (10♂/3♀) *Age:* 19.92 ± 1.75 y *Level:* NA (TL = 3.55 ± 1.44 sessions/week; TF = 6.72 ± 4.03 h/week; YoE = 10.80 ± 4.48 y; Exp = registered recreational leagues or higher)	Incongruent Stroop task (PC)	Congruent Stroop task	30 min	*S:* MF ↑ in I_2_ vs C_2_ and in I_2_ vs I_1_ (assessed using a 7-pt Likert scale) *B:* ↑ in RT in I vs C, no effect on ACC (assessed during the Stroop tasks)	**↓ in duration time (medium) in I vs C (measured using a beep test) (ES(η**^**2**^_**p**_**))**
Marcora et al. [78]	*N:* 16 subjects (10♂/6♀) *Age:* 26.00 ± 3.00 y *Mass:* 69.00 ± 10.00 kg *Height:* 175.00 ± 9.00 cm *Level:* PL2 (calc.) (PPO = 288.00 ± 70.00 W; VO2peak = 52.00 ± 8.00 ml/kg.min)	AX-CPT	Docu	90 min	*B:* ↓ in ACC (assessed during the AX-CPT) *P:* no effect on glucose (assessed using a Physioflow PF05L1)	**↓ in TTE (small) in I vs C, no effects on RPM (assessed using a TTE test) (ES(d))**
Martin et al. [31]	(1) Elite group (EG) *N:* 8 cyclists (♂) *Age:* 23.40 ± 6.40 y *Mass:* 68.20 ± 4.30 kg *Height:* 180.00 ± 7.00 cm *Level:* PL5 (PPO: 414.00 ± 48.00 W; TL > 5 sessions/week; TV > 500 km/week; YoE > 5y) (2) Non-elite group (NEG) *N:* 9 participants (♂) *Age*: 25.60 ± 5.30 y *Mass:* 80.70 ± 11.30 kg *Height:* 177.00 ± 7.00 cm *Level:* PL1/2 (PPO: 261.00 ± 28.00 W; TL ~ 3 sessions/week; TV ~ 80 km/week; YoE = 2 y)	Modified incongruent Stroop task (PC)	Sitting and focussing on a centred black cross	I: 30 min C: 10 min	*B:* ↓ in RT with no effects on ACC (assessed during the Stroop task)	**No evidence of MF based on manipulation checks**
O’Keeffe et al. [81]	*N:* 15 active participants (♂) *Age:* 24.00 ± 3.00 y	TDLoadDback	Resting	I: 16 min C: 2 min	*S:* MF ↑ in I_1_ vs I_2_ (assessed using VAS)	**No performance effects (PPO, APO and oxygen consumption) in I vs C (assessed using a time-based time trial)**
Pageaux et al. [72]	*N:* 12 adults (8♂/ 4♀) *Age:* 21.00 ± 1.00 y *Mass*: 69.00 ± 11.00 kg *Height:* 174.00 ± 12.00 cm *Level:* PL2 (TF ≥ 2 aerobic activities/week)	Modified incongruent Stroop task (PC)	Congruent Stroop task (PC)	30 min	*B:* ↑ in RT in I vs C, no effect on ACC (comparison of Stroop task responses) P: no effect on glucose (assessed using Biosen EFK Diagnostics)	**↑ in completion time (medium) and ↓ in running speed (medium) in I vs C, no effect on pacing (measured using a 5 km running time trial) (ES(η**^**2**^_**p**_**))**
Penna et al. [84]	*N:* 16 swimmers (11♂/ 5♀) *Age:* 15.45 ± 0.51 y *Level:* NA (TL = 30,000 m/week; YoE = 7.35 ± 2.20 y; Exp = competing in state and national competitions)	Stroop task (paper version)	Docu	30 min	*S:* MF ↑ in I_2_ vs I_1_ and in I vs C (assessed using M-VAS) P: no effect on HRV (assessed using a polar V800)	**↑ in completion time (trivial), ↓ in mean speed (trivial) and impaired pacing (trivial) in I vs C (measured using a 1500 m swimming task) (ES(d))**
Penna et al. [83]	*N:* 12 handball players (?) *Age:* 17.50 ± 3.63 y *Level:* NA (TL = ~ 6 h/week; YoE = 5.00 ± 2.20 y; Exp = regional level)	Stroop task (paper version)	Docu	30 min	*S:* MF ↑ in I_2_ vs I_1_ and in I_2_ vs C_2_ (assessed using M-VAS)	**↓ in total distance covered (small) in I vs C (measured using the Yo-Yo IR1) (ES(d))**
Pires et al. [80]	*N:* 8 cyclists (♂) *Age:* 29.30 ± 7.90 y *Mass:* 67.60 ± 7.50 kg *Height:* 177.20 ± 4.60 cm *Level:* PL3 (calc.) (PPO = 318.90 ± 22.40 W; VO_2_Max = 64.10 ± 4.80 ml/kg.min; YoE = 5.00 ± 3.20 y; Exp = competing at regional level)	RVIP test (PC)	Rest	30 min	*B:* ↑ in errors and ↓ in ACC, no effects on RT (assessed during RVP test) P: ↑ in θ PFC power in I_1,2,3_ vs C_1,2,3_ (blocks during RVP test) (assessed using EEG)	**↑ in time to completion (moderate) and ↓ in APO (moderate) in I vs C (measured using a cycling time trial) (ES(d))**
Salam et al. [73]	*N:* 11 cyclists (♂) *Age:* 38.00 ± 6.00 y *Mass:* 76.50 ± 9.60 kg Level: PL3 (calc.) (VO2peak = 60.50 ± 4.10 ml/kg.min; TL > 5 h of training/week; YoE ≥ 3y)	Modified Stroop task (PC)	Reading magazines	30 min	*S:* MF ↑ in I_2_ vs C_2_ (assessed using a 10 pt Likert scale)	**↓ in TTE (small to moderate) and estimated W’ (moderate) in I vs C, no effects on CP (measured using a TTE test) (ES(d))**
Schücker et al. [74]- study 1	*N:* 12 sport/endurance athletes (3♂, 9♀) *Age:* 29.41 ± 14.47 y *Level:* NA (TF = 5.87 ± 3.52 sessions/week; TL = 8.25 ± 4.31 h/week; YoE = 10.00 ± 7.35y)	Unmatched Stroop task (PC)	Matched Stroop task (PC)	10 min	*S:* MF ↑ in I vs C (assessed using a Likert scale) *B:* ↑ in RT and ↓ in ACC in I vs C (assessed during the Stroop task) *P:* no effect on glucose (assessed using Accu-Chek performa meter)	**No effects on running time in I vs C (measured using a beep test)**
Schücker et al. [74]- study 2	*N:* 14 sport/endurance athletes (5♂, 9♀) *Age:* 30.64 ± 13.11 y *Level:* NA (TF = 5.04 ± 2.77 sessions/week; TL = 7.89 ± 4.16 h/week; YoE = 14.54 ± 8.65y)	Unmatched Stroop task (PC)	Matched Stroop task (PC)	10 min	*S:* MF ↑ in I vs C (assessed using a Likert scale) *B:* ↑ in RT and ↓ in ACC in I vs C (assessed during the Stroop task)	**No effects on running time in I vs C (measured using a beep test)**
Slimani et al. [85]	*N:* 10 active endurance athletes (♂) *Age:* 16.00 ± 1.05 y *Mass:* 55.50 ± 4.20 kg *Height:* 1.62 ± 0.04 cm Level: PL1 (calculated based on performance parameters) (VO_2_ Max = 39.20 ± 4.80 ml/min/kg; Exp = “engaged in track events”)	Stroop task (paper version)	Reading magazines	30 min	*B:* ↓ in concentration performance and ↑ in errors in I_2_ vs C_2_ (assessed using the d2 test)	**↓ in estimated (moderate) and speed related (moderate) VO**_**2**_**Max values in I vs C (measured using a beep test) (ES(d))**
Smith et al. [86]	*N:* 12 soccer players (♂) *Age:* 24.00 ± 0.40 y *Mass:* 76.10 ± 2.00 kg *Height:* 175.30 ± 1.30 cm *Level:* NA (Exp = recreational)	Modified Stroop task (paper version)	Reading emotionally neutral magazines	30 min	*S:* MF ↑ in I_2_ vs C_2_ (assessed using M-VAS)	**↓ in total distance (small) (measured using the Yo-Yo IR1) (ES(d))**
Veness et al. [75]	*N:* 10 cricket players (♂) *Age:* 21.00 ± 8.00 y *Mass:* 77.10 ± 9.90 kg *Height:* 1.85 ± 0.08 m *Level:* NA (Exp = “elite”; YoE ≥ 2 y)	Modified Stroop task (PC)	Reading emotionally neutral magazines	30 × 60 s Stroop	S: MF ↑ in I vs C (assessed using M-VAS)	**↓ in total distance (small) (measured using the Yo-Yo IR1) (ES(d))**
Weerakoddy et al. [70]	*N:* 25 Australian football players (♂) *Age:* 23.80 ± 4.60 y *Level:* NA (Exp = community level)	Incongruent Stroop task (app)	Documentary	I: 35 min C: 30 min	*B:* ↓ in test score in I_1_ vs I_30_ (assessed during Stroop)	**↓ in total distance (small) (measured using the Yo-Yo IR1) (ES(d))**
Zering et al. [82]	*N:* 15 university students (8♀/7♂) *Age:* 19.56 ± 1.69 *Level:* PL 1 (Frequency of PA = 6.00 ± 2.00 sessions (> 10 min)/week)	Stop signal task (PC)	Documentary	10.5 min	S: MF ↑ in I_2_ vs C_2_ (assessed using ME Borg scale)	**↓ in PPO (medium) and VO**_**2**_**Peak (moderate) in I vs C (measured using a graded exhaustion test) (ES(η**^**2**^_**p**_** + d))**

Effect size scales (effect sizes were interpreted using the originally reported value of the literature):

Range of effect sizes (Cohen's d): < 0.2 = trivial; 0.2–0.6 = small; 0.6–1.2 = moderate; 1.2–2.0 = large; > 2.0 = very large

Range of effect size (Cohen's dz): < 0.2 = trivial; 0.2–0.5 = small; 0.5–0.8 = moderate; > 0.8 = large

Range of effect size (h^2^): < 0.25 = trivial; 0.25–0.5 = small; 0.5–1.0 = moderate; ≥ 1.0 = large

Range of effect size (η^2^): < 0.02 = trivial; 0.02–0.13 = small; 0.13–0.26 = moderate; > 0.26 = large

Range of effect size (η^2^_p_): ~ 0.01 = small; ~ 0.06 = medium; ≥ 0.1 = large

Range of effect size (SMD): < 0.2 = trivial; 0.2–0.5 = small; 0.5–0.8 = moderate; > 0.8 = large

♀ = female; ♂ = male; θ = theta, ACC = accuracy; APO = average power output; AX-CPT = AX continuous performance task; B = behavioural; C = control group (C1 = pre, C2 = post); calc = calculated based on given performance parameters; cm = centimeters; CP = critical power; d = Cohen’s d; Docu = documentary; EEG = electroencephalography; EG = elite group; ES = effect size; Exp = expertise; FINA points = points of the fédération internationale de natation; fNIRS = functional near infrared spectroscopy; h = hours; HRV = heart rate variability; I = intervention group (I1 = pre/first block, I2 = post/second/last block, I3 = third block,…); IAAF score = 2017 scoring tables of the international association of athletics federation; kg = kilogram; maxHR = maximal heart rate; ME = mental exertion; MF = mental fatigue; mg = milligrams; MIET = maximal incremental exercise test; min = minutes; ml = milliliters; M-VAS = mental visual analogue scale; NA = not available; NEG = non elite group; P = physiological; PA = physical activity; PA-R = physical activity rating tool; PC = portable computer; Plevel = performance level; PPO = peak power output; pt = point; RE = running economy; RI = response inhibition; RPM = revolutions per minute; RT = reaction time; RVIP = rapid visual information processing test; S = subjective; TF = training frequency; TL = training load; TTE = time to exhaustion; TV = training volume; VT = ventilatory threshold; W = watt; W’ = watt prime; W_get_ = rate of work that precipitated metabolic rate at gas exchange threshold; y = years; YoE = years of experience; YoY o IR1 test = Yo-Yo intermittent recovery test variant 1; η^2^ = eta squared

### Risk of Bias

**Fig. 3:**
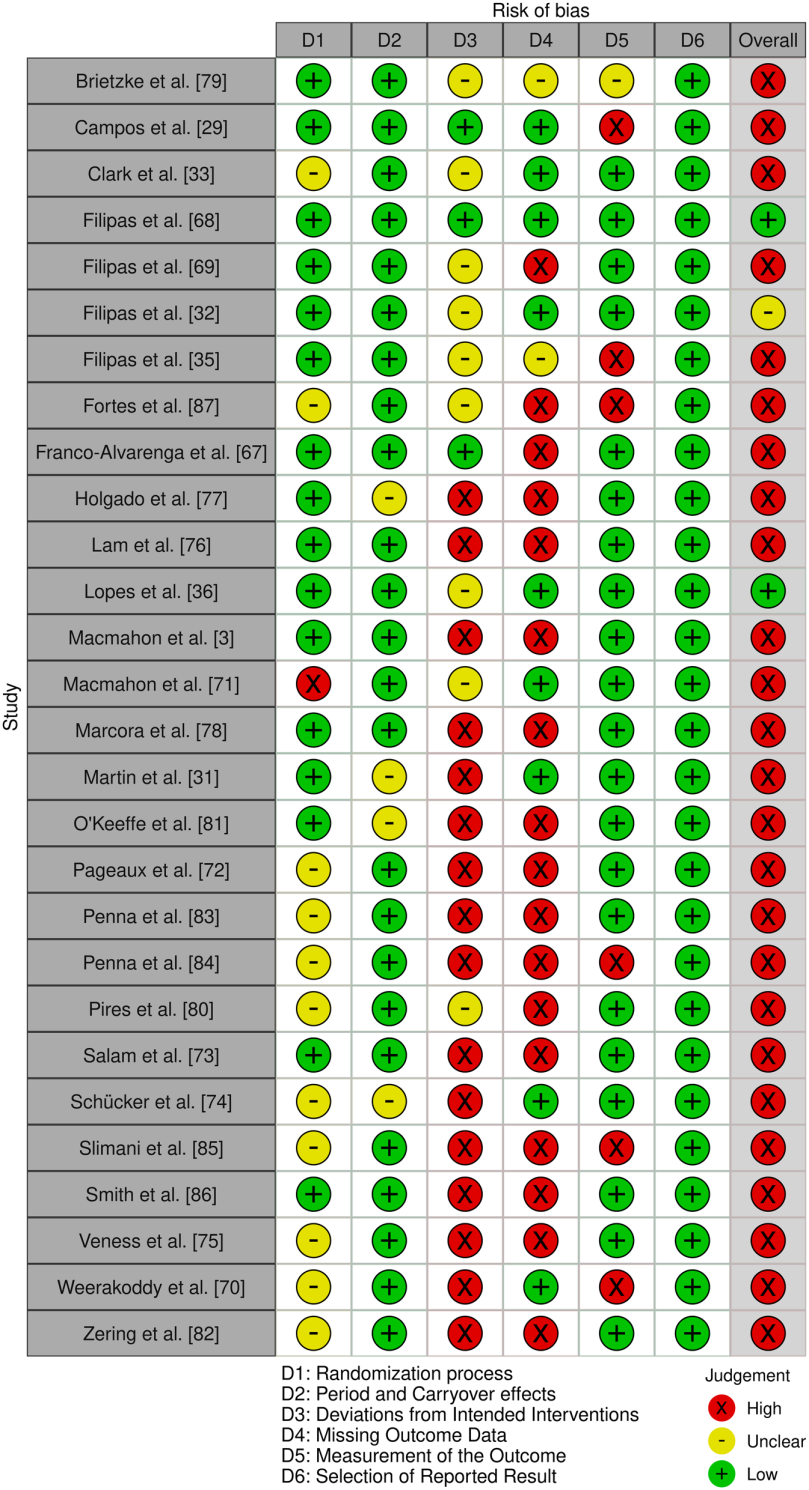
Risk of bias within studies

**Fig. 4:**
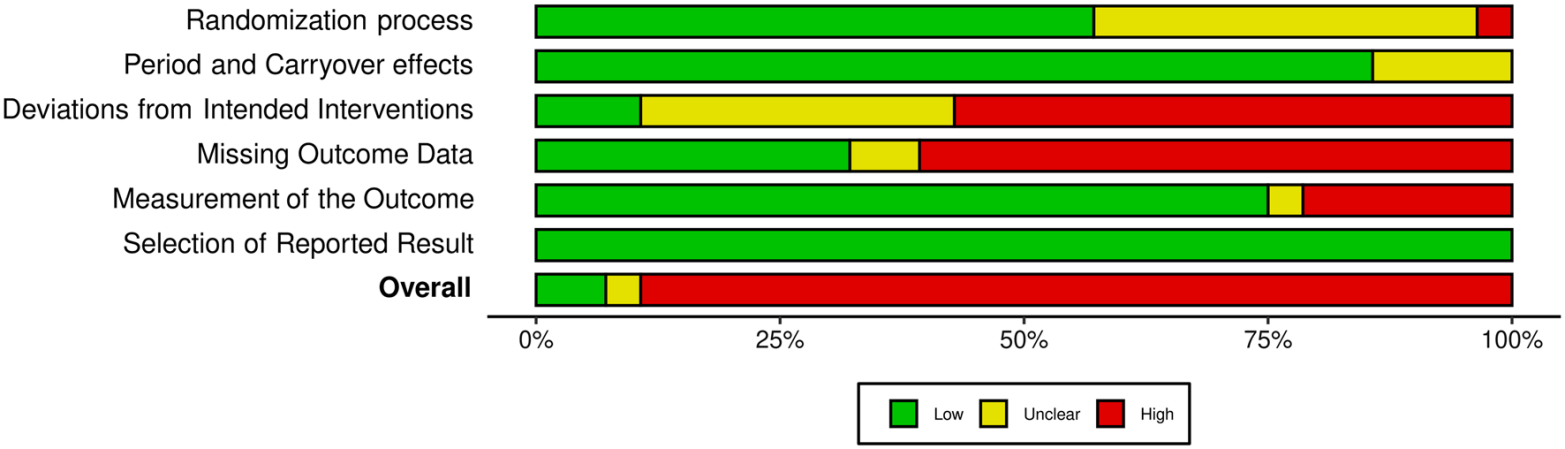
Risk of bias between studies

All but three of the included studies [32, 36, 68] featured a high risk of bias as determined by the RoB-2 tool for cross-over trials. The high risk of bias present in the other included studies was mainly the result of bias arising from deviations of intended interventions and bias due to missing outcome data. Figures 3 and 4 detail the RoB within and across studies, respectively.

### Mental Fatigue Characteristics

#### Interventions and Control Tasks

The following intervention tasks were used to induce MF: computer/app-based cognitive tasks (i.e., Stroop [31, 35, 36, 68–76], AX continuous performance task (AX-CPT) [3, 77, 78], rapid visual information processing test (RVIP) [67, 79, 80], TloadDback [81], stop-signal task [82] or a combination of cognitive tasks [32, 33]), paper-based cognitive tasks (i.e., Stroop [29, 83–86], an arithmetic task [68]) and smartphone use [87]. In most studies, some form of documentary movie was included as a control condition [3, 29, 33, 36, 69, 70, 77, 78, 82–84, 87]. Other control tasks consisted of drawing a mandala [68], reading (emotionally neutral) magazines [35, 73, 75, 85, 86], staring at a white screen [32] or black cross [31], performing cognitive tasks that required less cognitive load [71, 72, 74] and rest [80, 81]. Some studies employed no control tasks [67, 76, 79]. Duration of all interventions ranged between 10 [74] and 90 [3, 32, 77] min, with almost all studies applying the same duration for the intervention and control tasks, although there were some exceptions [32, 35].

#### Manipulation Checks

A variety of manipulation checks were used to evaluate the presence of MF in participants. These specific checks were either subjective (i.e., mental fatigue visual analogue scale (M-VAS) [29, 32, 35, 36, 67, 75–77, 79, 81, 83, 84, 86, 87], Likert scale [71, 73, 74] and Borg scale [82]), behavioural (i.e., cognitive performance on the intervention task [3, 31, 33, 35, 68–70, 72, 74, 77–81] or on a different pre-post cognitive task [36, 85, 87]) and/or physiological (i.e., electroencephalography [67, 79, 80], functional near-infrared spectroscopy [33] and heart rate variability [69]).

As with previous research [1], studies that showed no evidence of MF despite using appropriate manipulation checks (i.e., Clark et al. [33], Filipas et al. [68, 69], Macmahon et al. [3], and Martin et al. [31]) were excluded from further qualitative and quantitative analyses.

### Effects of Mental Fatigue on Dynamic Maximal Whole-Body Endurance Performance

#### Qualitative Overview of Included Studies

The following tasks were used as outcome measures: Time trials (*N* = 8 [32, 67, 72, 76, 80, 81, 84, 87]), time-to-exhaustion (TTE) task (*N* = 4 [36, 73, 77, 78]), maximal incremental exercise tasks (*N* = 2 [79, 82]), the Yo-Yo IR1 test (*N* = 6; [35, 70, 75, 76, 83, 86]), the beep test (*N* = 3 [71, 74, 85]), and a judo specific fitness test (*N* = 1 [29]).

Time trials were divided based on nature of the time trial-goal. This varied between overcoming a specific distance as fast as possible (distance-based), or covering as much distance as possible within a given time frame (time-based). One study [32] showed negative effects of MF using a time-based trial, showing a decrease in total distance. Contrarily, O’Keeffe et al. [81] found no effects of MF on average/maximal power output and oxygen consumption. Meanwhile, in the distance-based trials, all studies [67, 72, 76, 80, 84, 87] showed an increase in time to complete the trials when comparing mentally fatigued individuals with the control groups (although some supported small effects [76]), which they attributed to a negative effect on speed [72, 84], pacing [84] and average power output during the trial [67].

Three [36, 73, 78] out of the four studies examining TTE tests showed a significant negative effect of MF on test performance (i.e., decrease in TTE) compared to the control group. Lopes et al. [36] observed this negative effect in two distinct groups based on sex, but found no influence of this feature on the overall effect of MF on TTE performance. Salam et al. [73] even portrayed this effect on four different TTE tests based on VO_2_max percentage. In contrast, Holgado et al. [77] found no effect of MF on TTE performance.

Brietzke et al. [79] and Zering et al. [82] examined the effect of MF on a graded maximal exercise test. Both found a decrease in Peak Power Output (PPO) when comparing groups, which also resulted in a decrease of the TTE in the study of Brietzke et al. [79] (Zering et al. [82] did not report on TTE). VO_2_peak measures were also investigated in both studies, with Brietzke et al. [79] reporting no effect and Zering et al. [82] reporting a decrease in peak value. Zering et al. [82] also investigated a possible effect of sex on the responses to MF, but found no interaction between sex and condition.

All studies [35, 70, 75, 76, 83, 86] that utilized the Yo-Yo IR test required participants to perform the level 1 variant. This variant consists of two 20-m runs multiple times with an active recovery period in between [60]. The test is stopped if participants fail to reach the finish line on two consecutive occurrences, enforced by beeping sounds [60]. MF caused a decrease in total distance covered in all studies [35, 70, 75, 76, 83, 86]. Moreover, an effect of age was investigated by Filipas et al. [35], which illustrated a greater resilience to MF in younger players compared to the older ones.

The beep test is a well-known measure of basic physical performance, with instructions and test setup that are identical to the Yo-Yo IR1, but with no active recovery in-between bouts [61, 88]. Macmahon et al. [71] and Slimani et al. [85] both showed a negative influence of MF, with a decrease of total duration and of both estimated and speed-related VO_2_max values respectively, compared to the control condition. Schücker et al. [74] found no effect of MF on total beep test duration on two separate occasions.

Lastly, Campos et al. [29] examined the effect of MF on a judo specific fitness test (performing as many judo throws as possible in a given time frame [89]), showing no influence of MF on the total number of throws and performance index.

#### Overall Meta-analysis and Publication Bias

**Fig. 5:**
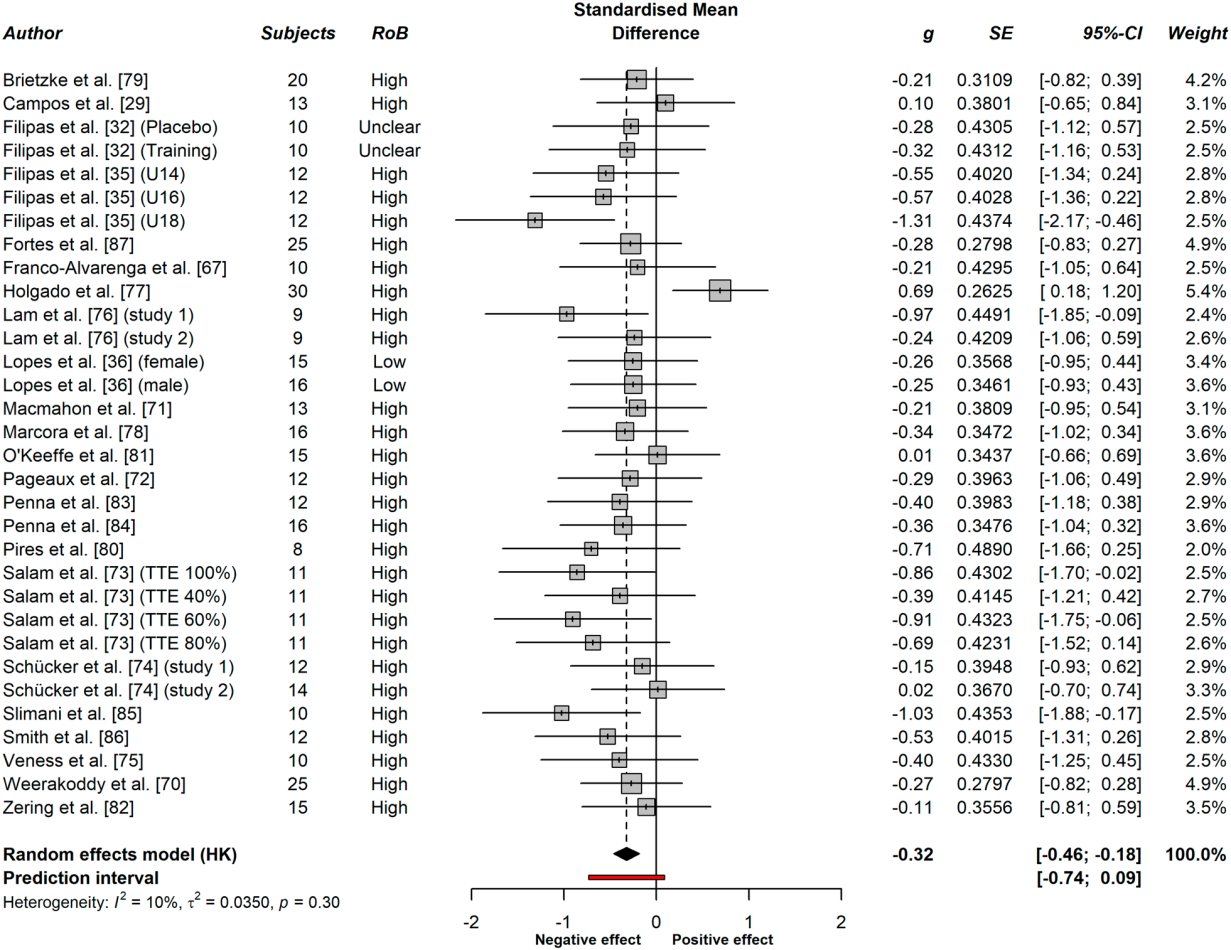
Forest plot detailing the effect of mental fatigue on dynamic maximal whole-body endurance performance (random effects meta-analysis; 95%-CI = 95% confidence interval; *g* = Hedges’ g; RoB = Risk of Bias; SE = standard error; TTE = time to exhaustion)

The forest plot of the overall effect of MF on endurance performance can be found in Fig. 5. Twenty-three studies were eventually included in the meta-analysis, which contributed to 32 distinct effects of MF (based on different groups/outcome measures within these studies) in 437 participants. The pooled effect across all studies was *g* = − 0.32 (95% CI [− 0.46; − 0.18], *t* = − 4.72, *p* < 0.0001). The test of heterogeneity was non-significant (*Q* = 34.52, *df* = 31, *p* = 0.3034). I^2^ was equal to 10.2%, with a confidence interval between 0.0 and 41.1%. The prediction interval ranged between − 0.74 and 0.09.

**Fig. 6:**
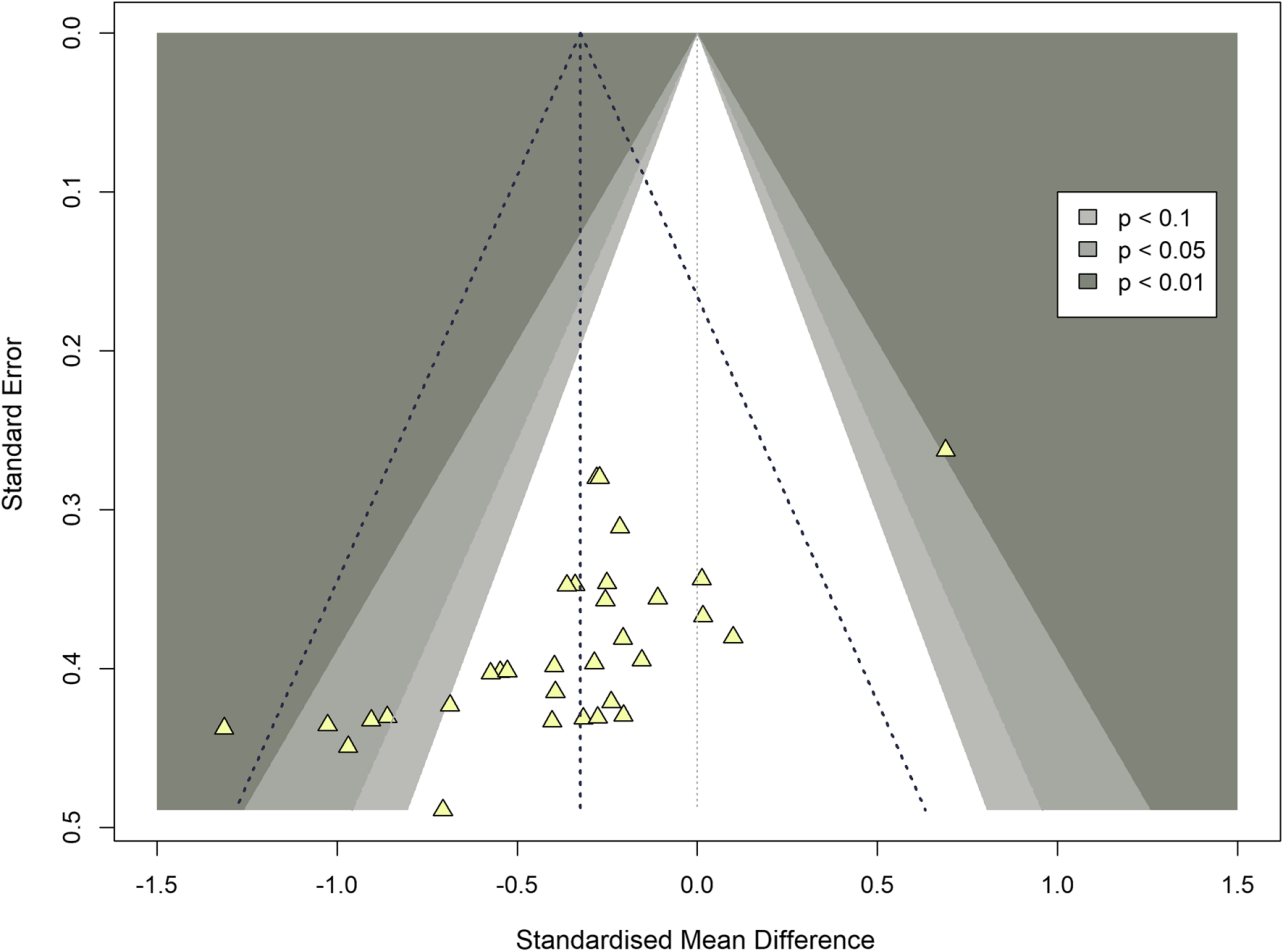
Funnel plot detailing publication bias of the present review (every study is represented by a yellow triangle; shades of grey detail the significance levels of the included studies; the triangle in the bold dashed line represents the effect of the present meta-analysis)

Egger’s test (*t* = -5.06, *df* = 31, *p* < 0.0001) showed the significant presence of publication bias, also seen in the funnel plot (Fig. 6). Rosenthal’s fail safe *N* was also significant (*p* < 0.0001), and indicated that 284 studies were needed to provide a null-effect for the overall meta-analysis.

### Influence of Different Features on the Effect of Mental Fatigue on Dynamic Maximal Whole-Body Endurance Performance

**Fig. 7:**
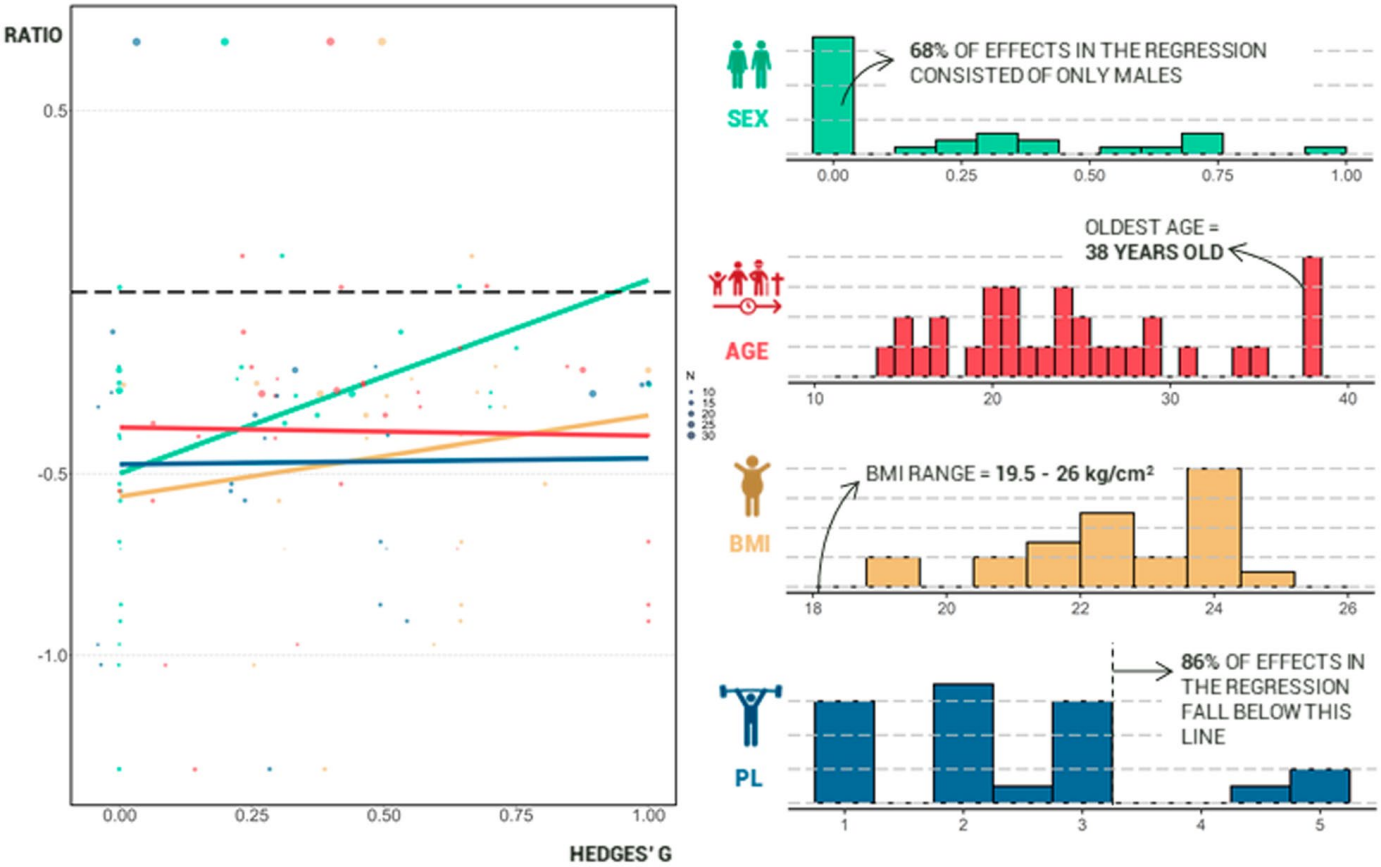
Visual representation of the regressions and the overall distribution of the included individual features. Ratios were calculated for all features except for sex to portray all equations in the same graph (ratio = value individual feature − inimal value of that feature/maximal value of that feature − minimal value of that feature). Sex ratio was calculated by dividing the total number of women by the total number of participants

Due to the underreporting of many of the investigated features of the included articles and to avoid overfitting, only four features were eventually chosen to be included in the analysis. These features included sex ratio, mean age, BMI (either given or calculated based on height and weight), and training level (either based on given data or calculated using the field-based performance test [58, 59]). Results showed no evidence of any significant influence of the included individual features on the proven negative effect of MF on endurance performance. The complete regression equation is presented below:

$y\left( {{\text{Hedges'}}\,g} \right) = 0.53x_{1} \left( {{\text{Sex}}} \right){-}0.003x_{2} \left( {{\text{Age}}} \right) + 0.05x_{3} \left( {{\text{BMI}}} \right) + 0.006x_{4} \left( {{\text{Performance}}\;{\text{level}}} \right) - 1.54$ The amount of residual heterogeneity consisted of 27.66%, while the model itself accounted for a heterogeneity of 18.97%. The test for residual heterogeneity was non-significant (*p* = 0.0609). Due to the omittance of individual features throughout the different manuscripts, 22 effects distributed across 15 studies [32, 35, 36, 67, 72, 73, 75–80, 85–87] were eventually included in this model. Attempts to improve the model (i.e., omitting the values that contributed least to the overall effect) resulted in a decrease of the estimates. As such, a better model could not be constructed. A visual representation of the regression can be found in Fig. 7.

An overview of the meta regression results is presented in Additional file 3.

## Discussion

The overall aim of this meta-analysis and meta-regression was to clarify the sometimes contradictory results regarding the effect of MF on endurance performance, by evaluating the contribution of different participant characteristics to MF-susceptibility. Based on the literature, we expected a negative effect of MF on endurance performance, which would be influenced by a combination of different individual features. The overall meta-analysis indeed confirmed a negative effect of MF on endurance performance. However, no influence was found of the individual features included in the multiple meta-regression on MF-related impairments in endurance performance. This review is therefore unable to confirm previous assumptions regarding the influence of previously investigated individual variables, such as physical fitness level, on MF-susceptibility.

### Effect of Mental Fatigue on Dynamic Maximal Whole-Body Endurance Performance

Unsurprisingly, the overall meta-analysis showed a significant negative effect of MF on endurance performance. The observed effect size was very similar to other published systematic reviews (e.g., Brown et al. [9] = − 0.26) that examined the link between MF and physical performance. However, these findings do contradict those found by recent/more critical studies. Holgado et al. [16] performed a bias sensitive meta-analysis and found a substantially lower and non-significant effect compared to other analyses [9]. Different multicentric studies further investigated these inconsistencies, and often found no clinically meaningful effect of MF on physical performance in larger sample sizes [17, 18, 90]. Multiple reasons can be put forward for this discrepancy. Most importantly, the heterogeneity in our review was very low, as a result of the focused inclusion criteria of the primary outcome measure. The results of other reviews that question effects of MF report higher heterogeneity, which might indicate that methodological factors (e.g. variety in primary outcome measure) played a role in their conclusions [16]. Secondly, the multicentric studies utilized a shorter duration of intervention tasks and a cognitive performance outcome, making comparison between both less straightforward [5]. Lastly, analyses show that, while there was significant publication bias present, 284 unpublished studies are necessary to completely nullify the effect of MF on endurance performance. This analysis therefore further solidifies the established effect of MF on endurance performance.

The CI of the current meta-analysis ranged between − 0.46 and − 0.18. Moreover, the prediction interval (i.e. the expected range of effects when a similar but novel study would be conducted, based on the results of past evidence [91]) showed a substantial range of effects (− 0.74 to 0.09). The present review therefore suggests that there is a large amount of variability in the way different individuals cope with the effects of MF, which confirms intuitive findings from the field regarding interindividual variability. At present, researching interindividual differences in MF-susceptibility remains one of the most important challenges in the MF research field. A first step in elucidating the mechanisms underlying these differences would be to determine whether they are stable and robust over time (i.e., trait-like) or whether they fluctuate over time (i.e., state-like). If the interindividual variability in MF-susceptibility has trait-characteristics, this would put forward a possible role for individual features (e.g., genes, age, physical fitness-level). While, if the interindividual differences in MF-susceptibility are determined to be state-like, this would suggest a possible role for other, more externally oriented, factors (e.g., sleep deprivation, mood). Since MF is seen as a multifactorial and complex phenomenon [4], the most likely explanation is that MF-susceptibility will be a dynamic combination of trait and state. More specifically, the individual factors that are featured in the analysis will probably play a part in how different persons cope with different state events to ultimately influence our level of MF throughout the day and its effects on performance. When the research field succeeds in identifying the nature of these differences, it will have opened a door to multiple future research opportunities, from further elucidating mechanisms of MF in general, to research lines linked to the detection, prevention and treatment of mental fatigue in practical settings. In the present review, we were unable to evaluate whether interindividual differences are robust over time. In an attempt to create additional insight in this matter, we performed a meta-regression that assessed the influence of often-reported participant characteristics in the effect of MF on endurance performance (i.e., physical fitness level, age, sex and BMI).

### Influence of Individual Features on Mental Fatigue-Susceptibility

#### Physical Fitness Level

Martin et al. [31] were the first authors to examine differences in MF-effects related to athlete proficiency by comparing MF-susceptibility between professional and recreational cyclists. Results showed that time trial performance of professional cyclists was not impacted by MF, compared to a decrease in performance in the recreational participants. This finding was attributed by Martin et al. [31] to the superior inhibitory control (i.e. the ability to stop, change, or delay an inappropriate response [92]) of the professional athletes (determined by comparing Stroop task performance). These results suggest that physical fitness could potentially help athletes to resist the negative effects of MF. Indeed, physical fitness-level has repeatedly been connected to improved cognitive functioning [32, 93–95]. Evidence for this interpretation can be found in a study of Filipas et al. [32], which indicated that a four week endurance training program increased the tolerance of initially untrained participants to MF. However, other studies and the current review have found no influence of training level on the effects of MF [33, 34, 96]. One explanation for this might be methodological, as there was a only small variation in physical fitness level for the effects included in the meta-regression, and some of the relevant variables were indirectly calculated. Another explanation is related to the complex interactions of different factors that determine the overall performance level of an athlete. For example, it could be that the resistance of elite cyclists to MF might be the result of talent identification and selection (i.e., the chance to become an elite athlete is higher in individuals that portray greater resistance to MF-effects) [31]. This highlights a possible influence of genetic [97, 98] and/or trait personality (e.g., hardiness, elite mentality) [39, 99] factors on MF resistance. Other features that could play a role in the way training level impacts MF-susceptibility include subject expertise [1, 100, 101], the person-situation fit [1], the type of sport (i.e. open or closed skill environment) [102], and the daily use of self-regulation [37]. Taken together, these findings show that, while the physical fitness level of participants might sometimes show up as a moderating variable, it is by no means the only parameter that needs to be considered when assessing influences of overall athlete training level on MF-susceptibility.

#### Age

Age is an important feature to consider as research clearly links increasing age with a degree of cognitive decline [54]. Physiological mechanisms behind this are connected to a decrease in neuronal glucose uptake, and increased neuro-inflammation and oxidative damage to the brain [103]. A psychological rationale behind a possible influence of older age on MF-susceptibility could be attributed to continuous changes in life events, goals and motivation [104, 105]. An effect of young age on MF-susceptibility is also possible. Research has found that younger individuals were more likely to have a decreased MF-susceptibility [35, 68]. This can possibly be linked to a decreased accessibility to brain regions that support complex behavior in young children compared to older ones, indicating a lower activation of complex cognitive processes and therefore limiting the impairments of these processes over time due to high cognitive load [35, 106]. Teenagers also display increased risk taking linked to a decrease in self-control, which might be driven by locally-connected subcortical regions [107]. While these regions are fully developed at an early stage, prefrontal cortex maturation persists until adulthood [107–109]. These findings might indicate an increase in MF-susceptibility when children reach adolescence, and a subsequent decrease again when they reach adulthood. However, similar to the review of Brahms et al. [40], the present model showed no influence of age on MF-susceptibility. That said, a major problem with the analyses is the limited range in age effects. In the present review, the oldest individuals were 38 years old, while the youngest were 14. Age-related declines in different cognitive functions and changes in life goals occur throughout the entire lifetime [54, 110], and a significant part of brain development also happens before the onset of adolescence [111]. Overall, it seems that effects of age on MF-susceptibility are primarily expected in either younger or older individuals than those primarily included in research (see also Fig. 7). This does not mean that researchers should resort to only testing children and elderly individuals, but rather that the current age range used in MF research is inadequate and limits the translation of research to the general population. Therefore, there is a large research potential present in the detailed investigation of this parameter, as the proven influence of age could have implications for both young as well as older individuals regarding the importance of MF screening and its effects on performance capacity [40].

#### Sex

Over the years, different studies have examined sex (i.e., the physiological/bodily aspect) and, to a lesser extent, gender (i.e., the societal norms aspect) differences in cognition and brain structure/activity [112–114]. For example, female participants regularly show improved performance in processing speed, while males have a faster reaction time [55, 112, 115, 116]. Xing et al. [117] identified 25 brain structures that were significantly different between men and women, and differences have been found in brain activation in the limbic system and the default mode network [118, 119]. It is argued that gender underlies fundamental differences in social cognition and societal expectations between different individuals, which could also impact the way that males and females cope with the effects of MF [113, 120]. However, our model showed that, while sex had the most substantial effect on MF responses, it did not reach significance. This is in line with other studies that have already used sex as a confounding feature in MF effects, with no influences of this feature found on the effects of MF on physical performance [36, 82] or on the subjective level of MF [116]. In the present model, the effect will likely have been influenced by the one-sided inclusion of male participants, increasing the negative effect on the far end of the ratio. This one-sided inclusion is a general finding in sport science literature, where female participants are often omitted from experimental investigations [121, 122]. The present review further confirms that sport-science research that focuses on including both males and females is urgently needed [121]. Additionally, because of the differences in social expectations among different genders in today’s society [113], assessing influences of sex and gender across different studies is very difficult to ascribe to either a more biological or a more psychological influence when examining different experiments. The influence of sex/gender on MF-susceptibility is therefore to be further elucidated.

#### BMI

Finally, BMI was also included in the model based on the participant characteristics that were mentioned by the included studies. BMI can be seen as potentially relevant as it is traditionally seen as a measure of general metabolic health [123]. Unhealthy nutrition and obesity have also been connected to decreases in cognitive performance and increases in cognitive decline and fatigue in diseased populations [56, 124, 125]. In healthy participants, BMI has been connected to decreased functional connectivity in brain areas related to cognition, cognitive flexibility and emotion regulation [126, 127]. However, the utilised model showed no influence of BMI on MF-effects, investigated utilizing a limited range of BMI values. This limited range is of course justifiable, since a very high or low BMI would classify participants as ‘diseased’, which was beyond the scope of this review. BMI has also been criticized for its extensive use as an indicator of biological health, as it seems to have a poor correlation with the percentage of body fat [128]. Therefore, in studies that do find differences in cognition linked to BMI, the real reason underlying these differences might be obscured. For example, research has shown that BMI is a proxy measure of socio-economic status [129], which also influences cognitive behavioural and structural outcomes [130]. This means that a potential influence of BMI on MF-susceptibility could also be the result of the societal standing of individuals. Therefore, studies that attempt to directly link BMI and MF-susceptibility might not be that relevant. Instead, a promising avenue for further research could lie in linking more appropriate measures of metabolic health (e.g., body fat percentage) to MF-susceptibility, clearing the way for novel countermeasures against MF.

#### Conclusions Based on the Meta Regression Model

The present model is restricted in its conclusions due to the limited reporting of different characteristics that influence cognitive functioning. The proposed features that might have an effect on MF-susceptibility were considered because they all influence the overall level of cognitive functioning. All integrated individual features (i.e. physical fitness level, age, sex, BMI) may contribute to improved or deteriorated cognitive functioning, which would allow people to receive increased cognitive load without becoming mentally fatigued, or ensure that performance is maintained while mentally fatigued. With this in mind, it is also important to understand that there is not one clear feature that definitively increases or decreases MF-susceptibility, leading to the possibility of a complex interaction between different features. An example supporting this theory can be seen in the study of Lopes et al. [36], which found no influence of sex on MF-susceptibility in highly trained individuals (performance level 5). This might mean that differences in MF-susceptibility based on sex are nullified by the high training level of participants. The interactions between different features and how these interactions influence MF-susceptibility warrant more extensive investigation.

When examining the included studies, it is clear that the current body of evidence examining MF-effects in sport science mainly includes physiological characteristics of participants. Studies that do investigate influences of one specific factor on MF-susceptibility also seem to focus on physiological features. While these features could definitely play a role, we must keep in mind that MF is defined as a psychobiological phenomenon. As such, a large number of individual features, namely those focusing on psychological characteristics, are currently predominantly omitted in research. It is understandable that sport scientists approach the research question from their own expertise and experience; in this case, physiological measurements. However, as mentioned before [4], the limited inclusion of the psychological perspective reduces the translatory value of MF research, including this review, and the impact it could have within the general research community. It is also important to know that the most sensitive indicators of MF are subjective measurements [131], which are arguably primarily influenced by the psychological state of the individual. Moreover, when all physiological features are similar in the same study population, it is likely that psychological constructs determine physical performance [132–134]. Features such as motivation [135] and perception of effort [136] have already been investigated in different research papers, but their true contribution to MF susceptibility still needs to be elucidated. Taken together, these findings suggest that psychological features have a similar or even larger influence on MF-susceptibility compared to the more often investigated physiological factors. For example, Martin et al. [37] showed that individuals who had higher levels of occupational cognitive demand were less affected by MF, implying that the level of self-regulation might be an important mediator in MF-susceptibility. Other examples of psychological features that could be included in future research include state/trait anxiety [137], trait self-control [138], mental toughness [139], motivation [135] and hardiness/resilience [39]. While these specific factors have already been investigated in some studies, more research is needed. It should be mentioned that these factors could have also impacted the results of the present study, as different performance tasks might trigger different levels of features such as motivation and perception of effort, which can be seen in the different pacing strategies that are triggered when comparing different performance tasks [140]. These changes could be the reason that no effects of individual features were found in the present analysis. In essence, this review further highlights that not only are there huge differences in MF-susceptibility across individuals, relevant features are currently not being extensively investigated.

To summarize, the utilized meta-regression model has primarily identified several gaps of knowledge in MF research, showing the need for high quality research examining changes in MF-susceptibility related to multiple different individual features.

### Proposed Guidelines for Future Research

The present review, in addition to the already published systematic reviews [1, 8, 9, 14, 16, 40, 45–50, 52, 141], confirms that MF impairs endurance performance. However, established assumptions on MF effects are questioned by the results of the present review (e.g., the notion that physically fit individuals are better at resisting MF compared to sedentary ones [31]). Therefore, while studies investigating effects of MF are important, an expanding focus on different promising research domains (such as interindividual differences) will further elucidate MF mechanisms and help in finding ways to counter and/or prevent it.

While this paper is the first attempt in elucidating the interindividual variation in responses to MF, the number of studies specifically examining these interindividual differences and especially their link with participant characteristics was very small. This gap in knowledge needs to be addressed by investigating the nature and occurrence of individual differences in MF-susceptibility in a truly holistic way, using knowledge of both exercise physiology and clinical psychology. Examples of features that warrant further investigation include sociodemographic variables (such as work situation [142]), baseline cognitive performance level (on cognitive domains such as attention [143] and working memory [144]), and different psychological determinants (see suggestions in *Conclusions Based on the Meta Regression Model*). Where it is already possible to do this, further systematic reviews should evaluate other features that might impact MF level and its effects, such as motivation [135] and perception of effort [136]. However, as mentioned, a systematic review is limited in its conclusions because of the large heterogeneity in performance outcomes, leading to differences in different psychological characteristics. Therefore, a large experimental trial investigating different individual's features in the same general population including one specific type of human performance should be the next step. Advanced knowledge on interindividual differences in MF-susceptibility and the features underlying these differences, has the potential to greatly augment our understanding of MF mechanisms. Moreover, determining possible underlying features also has great practical potential, from aiding in identifying individuals that are more susceptible to MF, to individualizing interventions that counter MF-effects [145].

The present review specifically investigated MF susceptibility, i.e. how much is performance affected when mentally fatigued. Consequently, articles that were considered for inclusion were required to have proof of a mentally fatigued state within their participants. However, another interesting research opportunity would be to assess the influence of different moderating variables on the induced level of MF, be it subjective, behavioural or neurophysiological. While out of the scope of the present review, future research should definitely consider further investigation along this line of research.

An important consideration is the lack of a detailed and thorough description of the individual characteristics of participants, especially when it concerns detailing the training/performance level of participants, as is seen across the sport science literature [146]. To define the performance level, an extensive number of different variables (e.g. VO_2_max, years of expertise, awards, level of play) should be taken into account. Different classifications of athlete performance already exist in the literature [58, 59, 146, 147], but are rarely used. Using these classifications will enhance the quality and comprehensiveness of results across sport science fields.

The authors of the present review chose to not exclude papers based on terminology, i.e., all possible equivalents of MF: mental fatigue, cognitive fatigue, self-control strength depletion and ego depletion were eligible for inclusion. Rather, the authors chose to include studies based on the presence of manipulation checks and the type of task used to induce MF. This methodology was applied, independently from the terminology-discussion, to increase the number of relevant articles that could be included in the meta-analysis. However, it is important that a consensus regarding the different interpretations of MF is reached. Different definitions, interpretations and terms that ultimately describe the same phenomenon are detrimental to interprofessional communication and act as a barrier to the development of this particular research field. This was, however, not the goal of the present review. Rather, this review should be interpreted as a call to action to finally agree on a consensus definition of MF, and the other representations in the literature.

### Limitations of the Present Review

The overall RoB of the included studies was high, with only three studies [32, 36, 68] detailing an unclear or low RoB. These ratings were primarily the result of high RoB values in the categories “deviations from the intended interventions”, and “missing outcome data”. These observations were also made by previous analyses [9]. Efforts should therefore be made to decrease the RoB in studies researching MF effects (e.g. blinding personnel, providing all information on the performed trial, etc.). Secondly, as with other reviews incorporating meta-analyses [9, 16], a significant amount of publication bias was present. However, Rosenthal's fail safe N showed that over 280 (un)published studies are necessary to completely nullify the found effect. While this measure has been criticized [148], it is highly unlikely that this amount of studies has remained unpublished. A recent meta-meta-analysis even suggested that the likeliness of publication is not that much higher for studies that document statistically significant results than those that do not [149]. The present review also included an abundance of studies whose effects failed to reach statistical significance. This shows that non-significant MF studies are being published, limiting the anticipated number of studies supporting null effects in gray literature. These limitations should be taken into account when interpreting the results of the present review.

## Conclusion

The present meta-analysis confirms the deleterious effect of MF on whole-body maximal dynamic endurance performance. Furthermore, it shows a large range of interindividual differences in MF-susceptibility. However, the included individual features (i.e. sex, age, BMI and physical fitness level) did not affect MF-susceptibility. The main reason for this might be mostly methodological and related to the poor reporting of the individual characteristics across the included studies. We thus conclude the main determinants of individual variations have not been adequately measured in the field so far. Therefore, this review primarily identifies substantial knowledge gaps within the MF research field.

## Supplementary Information

**Additional file 1.** Meta Analysis Data.

**Additional file 2.** R code used in the development of the meta analysis and regression.

**Additional file 3.** Meta regression result.

## Data Availability

All data generated or analysed during this study are included in this published article (and its supplementary information files).
