# Supplementary material for: Interindividual Variability in Mental Fatigue-Related Impairments in Endurance Performance: A Systematic Review and Multiple Meta-regression
Source: Sports Med Open. 2023 Feb 20;9:14. doi: 10.1186/s40798-023-00559-7 (PMC9941412; doi:10.1186/s40798-023-00559-7)
Supplement: Supplementary file 1 — Additional file 1. Meta Analysis Data. [file 40798_2023_559_MOESM1_ESM.pdf]

Supplementary Electronic Material 1: Meta Analysis Data

Article title:

Interindividual Variability in Mental Fatigue-Related Impairments in Endurance Performance: a Systematic Review and Multiple Meta-Regression

Journal name:

Sports Medicine Open

Author names:

Jelle Habay[1,2,3,4], Robin Uylenbroeck[1], Ruben Van Droogenbroeck[1], Jonas De Wachter[1,2], Matthias Proost[1,2], Bruno Tassignon[1,2], Kevin De Pauw[1,2], Romain Meeusen[1,2], Nathalie Pattyn[1,3], Jeroen Van Cutsem[1,3], Bart Roelands[1,2]

Affiliation:

[1] Human Physiology and Sports Physiotherapy Research Group, Faculty of Physical Education and Physiotherapy, Vrije Universiteit Brussel, Belgium.

[2] BruBotics, Vrije Universiteit Brussel, Brussels, Belgium

[3] Vital Signs and Performance Monitoring Research Unit, LIFE Department, Royal Military Academy, Brussels, Belgium

[4] Research Foundation Flanders (FWO), Brussels, Belgium

Corresponding author:

Prof. Dr. Bart Roelands; Faculty of Physical Education and Physiotherapy, Human Physiology and Sports Physiotherapy Research Group, Vrije Universiteit Brussel, Pleinlaan 2, 1050, Brussels, Belgium; bart.roelands@vub.be; 0032 2 629 28 75

Name	Year	Group	N	N_Men	N_Womer	Sex_ratio	Mean_Age	Mean_Weight	Mean_Height	BMI	Performance_Le	Physical performa	Outcome	RoB	M Int	SD int	M con	SD con	Within_groups_SD	Standardized_N	Variance_SMD	Correction_	Hedges_g	Variance_g	SE_g
Brietzke 2020	2020		20	20	0	0	35	80.5	176	26.04	2.5	MIT	TTE (sec)	High	827.75	68.62	841.85	59.65	64.29162815	-0.219313158	0.100601228	0.9801325	-0.214956	0.09664354	0.310875
Campos 2019	2019		13	9	4	0.307692	19.5	68.1	169	23.84370295	999	JFT	Throws (n)	High	25.8	1.9	25.6	2	1.95064092	0.102530403	0.154048317	0.9684211	0.0992926	0.14447257	0.380095
Filipas 2020-Training	2020	Training	10	3	7	0.7	27.6	69.6	169.4	24.25394719	1	Time trial	total distance (r	Unclear	6576.28	773.92	6822.43	715.36	745.2154373	-0.330307167	0.202727571	0.9577465	-0.316351	0.1859576	0.431228
Filipas 2020-Placebo	2020	Placebo	10	3	7	0.7	27.5	68.7	169.5	23.91207873	1	Time trial	total distance (r	Unclear	6561.79	681.43	6761.48	701.38	691.4769516	-0.288787644	0.202084958	0.9577465	-0.276585	0.18536815	0.430544
Filipas 2020-U14	2020	U14	12	12	0	0	13.92	55	168	19.48696145	2	Yo-Yo IR	total distance (r	High	1056.67	238.45	1203.33	277.37	258.6431126	-0.56703617	0.173365209	0.9655172	-0.547483	0.16161513	0.402014
Filipas 2020-U16	2020	U16	12	12	0	0	15.42	62	170	21.4532872	2	Yo-Yo IR	total distance (r	High	1090	357.01	1286.67	302.15	330.7194904	-0.594673147	0.174034086	0.9655172	-0.574167	0.16223867	0.402789
Filipas 2020-U18	2020	U18	12	12	0	0	17.33	69	177	22.02432251	2	Yo-Yo IR	total distance (r	High	1143.33	196.3	1400	180.5	188.5655589	-1.361171157	0.205266394	0.9655172	-1.314234	0.19135417	0.43744
Fortes 2020	2020		25	14	11	0.44	20.4	72	181	21.97735112	4.5	Time trial	completion tim	High	123.4	6.62	121.54	6.45	6.535552769	-0.284597197	0.080809956	0.9842932	-0.280127	0.07829136	0.279806
Franco-Alvarenga 2019	2019		10	10	0	0	34.30	77.60	179.30	24.14	3	Time trial	completion tim	High	1980	84	1962	84	84	-0.214285714	0.201147959	0.9577465	-0.205231	0.18450866	0.429545
Holgado 2019	2019		30	24	6	0.2	23.5	68.97	174.2	22.72814151	1	TTE	TTE (sec)	High	765.5	92.05	705.4	79.49	85.99960058	0.698840455	0.070736483	0.987013	0.6897646	0.0689111	0.262509
Lam 2021-Study1	2021	Study1	9	9	0	0	22	70	173	23.38868656	1	Yo-Yo IR	total distance (r	High	435.556	105.883	551.111	105.883	105.883	-1.091346108	0.255306565	0.8888889	-0.970085	0.20172371	0.449137
Lam 2021-Study2	2021	Study2	9	7	2	0.222222	21.1	74	179	23.09540901	999	Time trial	completion tim	High	1450.5	150	1408.5	162	156.115342	-0.269031855	0.224232726	0.8888889	-0.239139	0.17717154	0.420917
Lopes 2020-Male	2020	Male	16	16	0	0	25	63.2	172.7	21.19007057	5	TTE	TTE (sec)	Low	393.13	84.08	419.81	120.08	103.6548426	-0.257392702	0.126035172	0.9747899	-0.250904	0.11976056	0.346064
Lopes 2020-Female	2020	Female	15	0	15	1	25	52.4	163.8	19.53005616	5	TTE	TTE (sec)	Low	307.13	105.78	334.4	101.97	103.8924668	-0.262482939	0.134481622	0.972973	-0.255389	0.12731058	0.356806
Macmahon 2019	2019		13	10	3	0.230769	19.92	999	999	999	999	Beep test	duration time (s	High	528	152	560	148	150.0133327	-0.213314373	0.154721212	0.9684211	-0.206578	0.14510364	0.380925
Marcora 2009	2009		16	10	6	0.375	26.2	69	175	22.53061224	2	TTE	TTE (sec)	High	640	316	754	339	327.7018462	-0.347877198	0.126890915	0.9747899	-0.339107	0.1205737	0.347237
O'Keeffe 2021	2021		15	15	0	0	24	999	999	999	999	Time trial	Average power	High	84.773	21.070	84.505	20.475	20.77463026	0.01290035	0.133336107	0.9411765	0.0121415	0.11811088	0.343673
Pageaux 2014	2014		12	8	4	0.333333	21	69	174	22.79032897	2	Time trial	completion tim	High	1464	294	1386	228	263.0779352	-0.296490087	0.168498049	0.9655172	-0.286266	0.15707785	0.39633
Penna 2018-Swimmers	2018		16	11	5	0.3125	15.45	999	999	999	999	Time trial	completion tim	High	1377	76	1344	100	88.81441324	-0.371561313	0.127157153	0.9747899	-0.362194	0.12082668	0.347601
Penna 2018-Handball	2018		12	999	999	999	18	999	999	999	999	Yo-Yo IR	total distance	High	655.38	141.92	720	170.68	156.9601045	-0.411696974	0.1701978	0.9655172	-0.397501	0.1586624	0.398324
Pires 2018	2018		8	8	0	0	29.3	67.6	177.2	21.52877212	3	Time trial	completion tim	High	2058	78	2004	66	72.24956747	-0.747409319	0.267456897	0.9454545	-0.706642	0.23907552	0.488953
Salam 2018-TTE 40%	2018	TTE40%	11	11	0	0	38	76.5	179.6	23.71640022	3	TTE40%	TTE (sec)	High	648	171	720	180	175.5576828	-0.410121613	0.185640903	0.9620253	-0.394547	0.1718093	0.414499
Salam 2018-TTE 60%	2018	TTE60%	11	11	0	0	38	76.5	179.6	23.71640022	3	TTE60%	TTE (sec)	High	341	84	422	88	86.02325267	-0.941605874	0.201968673	0.9620253	-0.905849	0.18692053	0.432343
Salam 2018-TTE 80%	2018	TTE80%	11	11	0	0	38	76.5	179.6	23.71640022	3	TTE80%	TTE (sec)	High	231	65	275	58	61.59951299	-0.714291362	0.193413912	0.9620253	-0.687166	0.17900317	0.423088
Salam 2018-TTE 100%	2018	TTE100%	11	11	0	0	38	76.5	179.6	23.71640022	3	TTE100%	TTE (sec)	High	156	38	190	38	38	-0.894736842	0.200012591	0.9620253	-0.860759	0.1851102	0.430244
Schücker 2016-study 1	2016		12	3	9	0.75	29.41	999	999	999	999	Beep test	duration time (s	High	540	99	555	88	93.66162501	-0.160150969	0.167201007	0.9655172	-0.154629	0.15586872	0.394802
Schücker 2016-study 2	2016		14	5	9	0.642857	30.64	999	999	999	999	Beep test	duration time (s	High	509	125	507	121	123.0162591	0.016258013	0.142861863	0.9708738	0.0157845	0.13466101	0.366962
Slimani 2018	2018		10	10	0	0	16	55.5	162	21.1476909	1	Beep test	estimated VO2I	High	33.8	4.7	39.2	4.8	4.750263151	-1.136779128	0.23230667	0.9032258	-1.026768	0.1895197	0.435339
Smith 2015	2015		12	12	0	0	24	76.1	175.3	24.76400167	2	Yo-Yo IR	total distance (r	High	1203	402	1410	354	378.7611385	-0.546518581	0.17288922	0.9655172	-0.527673	0.1611714	0.401462
Veness 2017	2017		10	10	0	0	21	77.1	184.9	22.55176596	2	Yo-Yo IR	total distance (r	High	1732	402	1892	357	380.1664109	-0.420868323	0.204428254	0.9577465	-0.403085	0.1875176	0.433033
Weerakoddy 2021	2021		25	25	0	0	23.8	999	999	999	999	Yo-Yo IR	total distance (r	High	1040	492.75	1182.4	537.78	515.7566727	-0.276099191	0.080762308	0.9842932	-0.271763	0.0782452	0.279723
Zering 2017	2017		15	7	8	0.533333	19.56	999	999	999	1	MIT	PPO (W)	High	240.03	53.37	246.03	52.6	52.98639873	-0.113236607	0.133547042	0.972973	-0.110176	0.12642583	0.355564
