# Supplementary material for: Interindividual Variability in Mental Fatigue-Related Impairments in Endurance Performance: A Systematic Review and Multiple Meta-regression
Source: Sports Med Open. 2023 Feb 20;9:14. doi: 10.1186/s40798-023-00559-7 (PMC9941412; doi:10.1186/s40798-023-00559-7)
Supplement: Supplementary file 2 — Additional file 2. R code used in the development of the meta analysis and regression. [file 40798_2023_559_MOESM2_ESM.pdf]

Supplementary Electronic Material 2: R code

Article title:

Interindividual Variability in Mental Fatigue-Related Impairments in Endurance Performance: a Systematic Review and Multiple Meta-Regression

Journal name:

Sports Medicine Open

Author names:

Jelle Habay[1,2,3,4], Robin Uylenbroeck[1], Ruben Van Droogenbroeck[1], Jonas De Wachter[1,2], Matthias Proost[1,2], Bruno Tassignon[1,2], Kevin De Pauw[1,2], Romain Meeusen[1,2], Nathalie Pattyn[1,3], Jeroen Van Cutsem[1,3], Bart Roelands[1,2]

Affiliation:

[1] Human Physiology and Sports Physiotherapy Research Group, Faculty of Physical Education and Physiotherapy, Vrije Universiteit Brussel, Belgium.

[2] BruBotics, Vrije Universiteit Brussel, Brussels, Belgium

[3] Vital Signs and Performance Monitoring Research Unit, LIFE Department, Royal Military Academy, Brussels, Belgium

[4] Research Foundation Flanders (FWO), Brussels, Belgium

Corresponding author:

Prof. Dr. Bart Roelands; Faculty of Physical Education and Physiotherapy, Human Physiology and Sports Physiotherapy Research Group, Vrije Universiteit Brussel, Pleinlaan 2, 1050, Brussels, Belgium; bart.roelands@vub.be; 0032 2 629 28 75

Supplementary Material B

Interindividual Variability in Mental Fatigue-Related Impairments in Endurance Performance: a Systematic Review and Multiple Meta-Regression: R code

Jelle Habay

16-6-2022

Introduction

The following r markdown file contains all code chunks relevant to perform the meta-analysis and meta-regression seen in Habay et al. 2022, “Interindividual Variability in Mental Fatigue-Related Impairments in Endurance Performance: a Systematic Review and Multiple Meta-Regression”.

Questions on the content of the present document are to be send to the corresponding author: bart.roelands@vub.be

Preparation

Installation of necessary packages

Packages necessary for installation are:

- meta (https://cran.r-project.org/web/packages/meta/index.html)
- metafor (https://cran.r-project.org/web/packages/metafor/index.html)
- tidyverse (https://cran.r-project.org/web/packages/tidyverse/index.html)
- readxl (https://cran.r-project.org/web/packages/readxl/index.html).

Loading of necessary packages

```
library("meta")
library("metafor")
library("tidyverse")
library("readxl")
library("PerformanceAnalytics")
```

Loading of Basic MA file (with replacement of missing values)

```
BasicMAfile <- read_excel("SMA_Habay2022_MFIND_MA.xlsx")
BasicMAfile$N_Men[BasicMAfile$N_Men==999] <- NA
BasicMAfile$N_Women[BasicMAfile$N_Women==999] <- NA
BasicMAfile$Sex_ratio[BasicMAfile$Sex_ratio==999] <- NA
BasicMAfile$Mean_Age[BasicMAfile$Mean_Age==999] <- NA
BasicMAfile$Mean_Weight[BasicMAfile$Mean_Weight==999] <- NA
BasicMAfile$Mean_Height[BasicMAfile$Mean_Height==999] <- NA
BasicMAfile$BMI[BasicMAfile$BMI==999] <- NA
BasicMAfile$Performance_Level[BasicMAfile$Performance_Level==999] <- NA
glimpse(BasicMAfile)
```

```
## Rows: 32
```

```
## Columns: 26
## $ Name                               <chr> "Brietzke 2020", "Campos 2019", "Fil~
## $ Year                               <dbl> 2020, 2019, 2020, 2020, 2020, 2020, ~
## $ Group                              <chr> NA, NA, "Training", "Placebo", "U14"~
## $ N                                  <dbl> 20, 13, 10, 10, 12, 12, 12, 25, 10, ~
## $ N_Men                              <dbl> 20, 9, 3, 3, 12, 12, 12, 14, 10, 24,~
## $ N_Women                            <dbl> 0, 4, 7, 7, 0, 0, 0, 11, 0, 6, 0, 2,~
## $ Sex_ratio                          <dbl> 0.0000000, 0.3076923, 0.7000000, 0.7~
## $ Mean_Age                           <dbl> 35.00, 19.50, 27.60, 27.50, 13.92, 1~
## $ Mean_Weight                        <dbl> 80.50, 68.10, 69.60, 68.70, 55.00, 6~
## $ Mean_Height                        <dbl> 176.0, 169.0, 169.4, 169.5, 168.0, 1~
## $ BMI                                <dbl> 26.04000, 23.84370, 24.25395, 23.912~
## $ Performance_Level                  <dbl> 2.5, NA, 1.0, 1.0, 2.0, 2.0, 2.0, 4.~
## $ `Physical performance task`        <chr> "MIT", "JFT", "Time trial", "Time tr~
## $ Outcome                            <chr> "TTE (sec)", "Throws (n)", "total di~
## $ RoB                                <chr> "High", "High", "Unclear", "Unclear"~
## $ `M Int`                            <dbl> 827.750, 25.800, 6576.280, 6561.790,~
## $ `SD int`                           <dbl> 68.620, 1.900, 773.920, 681.430, 238~
## $ `M con`                            <dbl> 841.850, 25.600, 6822.430, 6761.480,~
## $ `SD con`                           <dbl> 59.650, 2.000, 715.360, 701.380, 277~
## $ Within_groups_SD                   <dbl> 64.291628, 1.950641, 745.215437, 691~
## $ `Standardized_Mean_difference(d)` <dbl> -0.21931316, 0.10253040, -0.33030717~
## $ Variance_SMD                       <dbl> 0.10060123, 0.15404832, 0.20272757, ~
## $ `Correction_factor(J)`            <dbl> 0.9801325, 0.9684211, 0.9577465, 0.9~
## $ Hedges_g                           <dbl> -0.21495594, 0.09929260, -0.31635053~
## $ Variance_g                         <dbl> 0.09664354, 0.14447257, 0.18595760, ~
## $ SE_g                               <dbl> 0.3108754, 0.3800955, 0.4312280, 0.4~
```

```
str(BasicMAfile)
```

```
## tibble [32 x 26] (S3: tbl_df/tbl/data.frame)
##  $ Name                            : chr [1:32] "Brietzke 2020" "Campos 2019" "Filipas 20
20-Training" "Filipas 2020-Placebo" ...
##  $ Year                            : num [1:32] 2020 2019 2020 2020 2020 ...
##  $ Group                           : chr [1:32] NA NA "Training" "Placebo" ...
##  $ N                               : num [1:32] 20 13 10 10 12 12 12 25 10 30 ...
##  $ N_Men                           : num [1:32] 20 9 3 3 12 12 12 14 10 24 ...
##  $ N_Women                         : num [1:32] 0 4 7 7 0 0 0 11 0 6 ...
##  $ Sex_ratio                       : num [1:32] 0 0.308 0.7 0.7 0 ...
##  $ Mean_Age                        : num [1:32] 35 19.5 27.6 27.5 13.9 ...
##  $ Mean_Weight                     : num [1:32] 80.5 68.1 69.6 68.7 55 ...
##  $ Mean_Height                     : num [1:32] 176 169 169 170 168 ...
##  $ BMI                             : num [1:32] 26 23.8 24.3 23.9 19.5 ...
##  $ Performance_Level               : num [1:32] 2.5 NA 1 1 2 2 2 4.5 3 1 ...
##  $ Physical performance task       : chr [1:32] "MIT" "JFT" "Time trial" "Time trial" ...
##  $ Outcome                         : chr [1:32] "TTE (sec)" "Throws (n)" "total distance
(m)" "total distance (m)" ...
##  $ RoB                             : chr [1:32] "High" "High" "Unclear" "Unclear" ...
##  $ M Int                           : num [1:32] 827.8 25.8 6576.3 6561.8 1056.7 ...
##  $ SD int                          : num [1:32] 68.6 1.9 773.9 681.4 238.4 ...
##  $ M con                           : num [1:32] 841.9 25.6 6822.4 6761.5 1203.3 ...
##  $ SD con                          : num [1:32] 59.6 2 715.4 701.4 277.4 ...
##  $ Within_groups_SD                : num [1:32] 64.29 1.95 745.22 691.48 258.64 ...
##  $ Standardized_Mean_difference(d): num [1:32] -0.219 0.103 -0.33 -0.289 -0.567 ...
##  $ Variance_SMD                    : num [1:32] 0.101 0.154 0.203 0.202 0.173 ...
##  $ Correction_factor(J)            : num [1:32] 0.98 0.968 0.958 0.958 0.966 ...
```

```
##  $ Hedges_g                      : num [1:32] -0.215 0.0993 -0.3164 -0.2766 -0.5475 ...
##  $ Variance_g                    : num [1:32] 0.0966 0.1445 0.186 0.1854 0.1616 ...
##  $ SE_g                          : num [1:32] 0.311 0.38 0.431 0.431 0.402 ...
```

Effect of Mental Fatigue on Dynamic Maximale Whole-Body Endurance Performance: a Meta-Analysis

Construction of Basic Meta-Analysis

```
REMA <- metagen(TE = Hedges_g,
                    seTE = SE_g,
                    studlab = Name,
                    data = BasicMAfile,
                    sm = "SMD",
                    fixed = FALSE,
                    random = TRUE,
                    method.tau = "REML",
                    n.e = N,
                    hakn = TRUE,
                    title = "Overall effect of Mental Fatigue on Whole Body Dynamic Enduran
ce Performance (Random)",
                    keepdata = TRUE)
summary(REMA)
```

```
## Review:     Overall effect of Mental Fatigue on Whole Body Dynamic Endurance ...
##
##                             SMD             95%-CI %W(random)
## Brietzke 2020          -0.2150 [-0.8243;  0.3943]        4.2
## Campos 2019             0.0993 [-0.6457;  0.8443]        3.1
## Filipas 2020-Training -0.3164 [-1.1615;  0.5288]        2.5
## Filipas 2020-Placebo   -0.2766 [-1.1204;  0.5673]        2.5
## Filipas 2020-U14       -0.5475 [-1.3354;  0.2404]        2.8
## Filipas 2020-U16       -0.5742 [-1.3636;  0.2153]        2.8
## Filipas 2020-U18       -1.3142 [-2.1716; -0.4569]        2.5
## Fortes 2020            -0.2801 [-0.8285;  0.2683]        4.9
## Franco-Alvarenga 2019 -0.2052 [-1.0471;  0.6367]        2.5
## Holgado 2019            0.6898 [ 0.1753;  1.2043]        5.4
## Lam 2021-Study1        -0.9701 [-1.8504; -0.0898]        2.4
## Lam 2021-Study2        -0.2391 [-1.0641;  0.5858]        2.6
## Lopes 2020-Male        -0.2509 [-0.9292;  0.4274]        3.6
## Lopes 2020-Female      -0.2554 [-0.9547;  0.4439]        3.4
## Macmahon 2019          -0.2066 [-0.9532;  0.5400]        3.1
## Marcora 2009           -0.3391 [-1.0197;  0.3415]        3.6
## O'Keeffe 2021           0.0121 [-0.6614;  0.6857]        3.6
## Pageaux 2014           -0.2863 [-1.0631;  0.4905]        2.9
## Penna 2018-Swimmers    -0.3622 [-1.0435;  0.3191]        3.6
## Penna 2018-Handball    -0.3975 [-1.1782;  0.3832]        2.9
## Pires 2018             -0.7066 [-1.6650;  0.2517]        2.0
## Salam 2018-TTE 40%     -0.3945 [-1.2070;  0.4179]        2.7
## Salam 2018-TTE 60%     -0.9058 [-1.7532; -0.0585]        2.5
## Salam 2018-TTE 80%     -0.6872 [-1.5164;  0.1421]        2.6
## Salam 2018-TTE 100%    -0.8608 [-1.7040; -0.0175]        2.5
## Schücker 2016-study 1 -0.1546 [-0.9284;  0.6192]        2.9
## Schücker 2016-study 2  0.0158 [-0.7034;  0.7350]        3.3
## Slimani 2018           -1.0268 [-1.8800; -0.1735]        2.5
## Smith 2015             -0.5277 [-1.3145;  0.2592]        2.8
```

```
## Veness 2017           -0.4031 [-1.2518;  0.4456]        2.5
## Weerakoddy 2021       -0.2718 [-0.8200;  0.2765]        4.9
## Zering 2017           -0.1102 [-0.8071;  0.5867]        3.5
##
## Number of studies combined: k = 32
## Number of observations: o = 437
##
##                          SMD             95%-CI     t  p-value
## Random effects model -0.3246 [-0.4650; -0.1843] -4.72 < 0.0001
##
## Quantifying heterogeneity:
##  tau^2 = 0.0350 [0.0000; 0.1135]; tau = 0.1870 [0.0000; 0.3370]
##  I^2 = 10.2% [0.0%; 41.1%]; H = 1.06 [1.00; 1.30]
##
## Test of heterogeneity:
##      Q d.f. p-value
##  34.52   31  0.3034
##
## Details on meta-analytical method:
## - Inverse variance method
## - Restricted maximum-likelihood estimator for tau^2
## - Q-profile method for confidence interval of tau^2 and tau
## - Hartung-Knapp adjustment for random effects model
```

Construction of Forest Plot

```
ForestplotRema <- forest.meta(REMA,
                              sortvar = studlab,
                              lty.random = 2,
                              comb.random = TRUE,
                              prediction = TRUE,
                              xlim = c(-2.5, 2.5),
                              lwd = 1.5,
                              label.e = "Effect Sizes",
                              label.e.attach = "g",
                              label.left = "Negative effect",
                              label.right = "Positive effect",
                              leftcols = c("studlab", "N", "RoB"),
                              leftlabs = c("Author", "Subjects", "RoB"),
                              rightcols = c("effect", "seTE", "ci", "w.random"),
                              rightlabs = c("g", "SE", "95%-CI", "Weight"),
                              col.square.lines = "black",
                              col.diamond = "black",
                              hetlab = "Heterogeneity: ",
                              fontsize = 11,
                              fs.lr = 9,
                              ff.heading = 4,
                              ff.lr = 2,
                              squaresize = 0.9,
                              plotwidth = unit(10, "cm"),
                              colgap = unit(5, "mm"),
                              colgap.forest.left = unit(1,"mm"),
                              colgap.forest.right = unit(1,"mm"),
                              addrow.overall = TRUE,
                              digits.pval = 4)
```

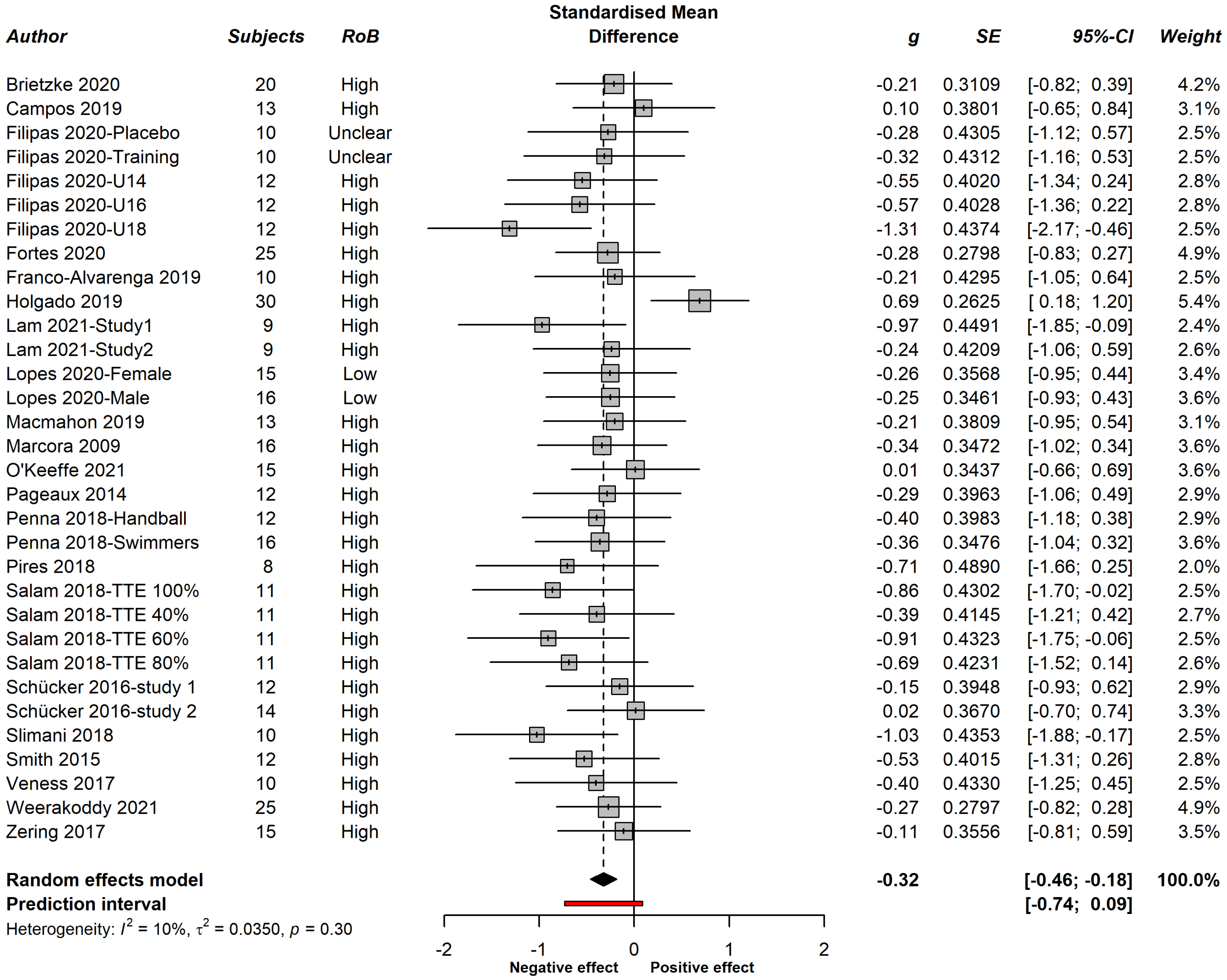

Examination of publication bias and construction of funnel plot

Contour enhanced funnel plot

```
col.contour = c("#B9BAB5", "#A5A7A0", "#818479")

FunnelPlotREMA <- funnel.meta(REMA,
                              xlim = c(-1.5,1.5),
                              pch = 24,
                              cex = 1.3,
                              lty.random = 3,
                              lwd.random = 2,
                              col = "black",
                              bg = c("#F6FEAA"),
                              col.random = c("#2D3047"),
                              studlab = FALSE,
                              contour = c(0.9, 0.95, 0.99),
                              col.contour = col.contour,
                              ref.triangle = TRUE,
                              lty.ref = 3,
```

```
                              col.ref = "grey65")

LegendFunnelPlotREMA <- legend(x = 1, y = 0.1,
                               legend = c("p < 0.1", "p < 0.05", "p < 0.01"),
                               fill = col.contour,
                               y.intersp = 1.5)
```

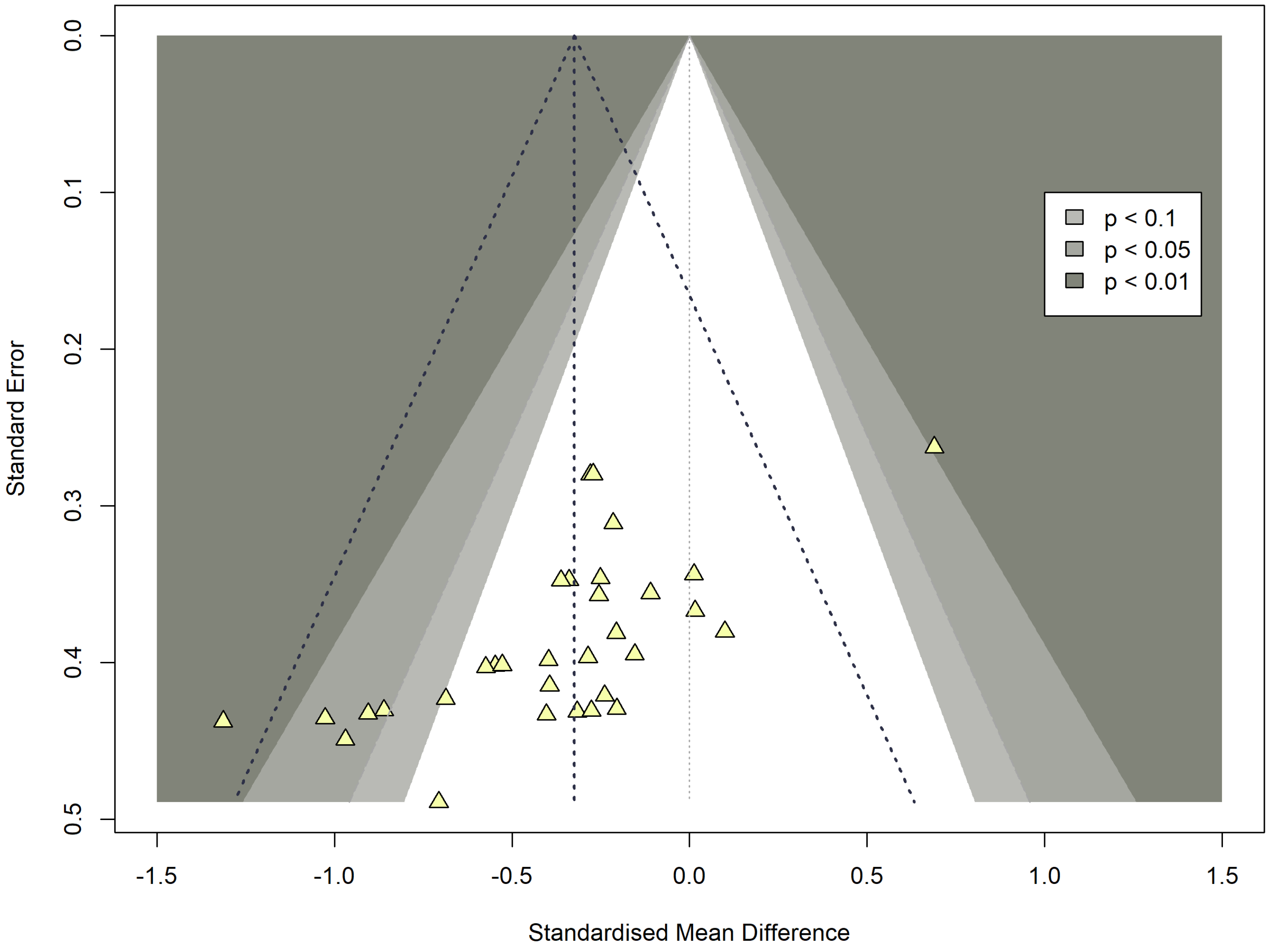

Egger’s regression test

```
metabias(REMA, method.bias = "linreg")
```

```
## Review:     Overall effect of Mental Fatigue on Whole Body Dynamic Endurance ...
##
## Linear regression test of funnel plot asymmetry
##
## Test result: t = -4.90, df = 30, p-value < 0.0001
##
## Sample estimates:
##     bias se.bias intercept se.intercept
##  -4.4730  0.9125    1.3517       0.3423
##
## Details:
## - multiplicative residual heterogeneity variance (tau^2 = 0.6389)
```

```
## - predictor: standard error
## - weight:    inverse variance
## - reference: Egger et al. (1997), BMJ
```

Rosenthal's fail safe N

```
fsn(yi = Hedges_g,
    sei = SE_g,
    data = BasicMAfile,
    type = "Rosenthal",
    alpha = 0.05)
```

```
##
## Fail-safe N Calculation Using the Rosenthal Approach
##
## Observed Significance Level: <.0001
## Target Significance Level:   0.05
##
## Fail-safe N: 284
```

Meta-Regression of Individual Features

Checking Multi-Collinearity

```
MCMR <- BasicMAfile[,c("Sex_ratio", "Mean_Age", "BMI", "Performance_Level")]
glimpse(MCMR)
```

```
## Rows: 32
## Columns: 4
## $ Sex_ratio         <dbl> 0.0000000, 0.3076923, 0.7000000, 0.7000000, 0.000000~
## $ Mean_Age          <dbl> 35.00, 19.50, 27.60, 27.50, 13.92, 15.42, 17.33, 20.~
## $ BMI               <dbl> 26.04000, 23.84370, 24.25395, 23.91208, 19.48696, 21~
## $ Performance_Level <dbl> 2.5, NA, 1.0, 1.0, 2.0, 2.0, 2.0, 4.5, 3.0, 1.0, 1.0~
```

```
cor(x = MCMR, use = "complete.obs")
```

```
##                     Sex_ratio    Mean_Age        BMI Performance_Level
## Sex_ratio          1.00000000 -0.08565502 -0.1729632         0.1058846
## Mean_Age          -0.08565502  1.00000000  0.6267302         0.2782959
## BMI               -0.17296324  0.62673024  1.0000000        -0.2953938
## Performance_Level  0.10588465  0.27829594 -0.2953938         1.0000000
```

```
chart.Correlation(MCMR)
```

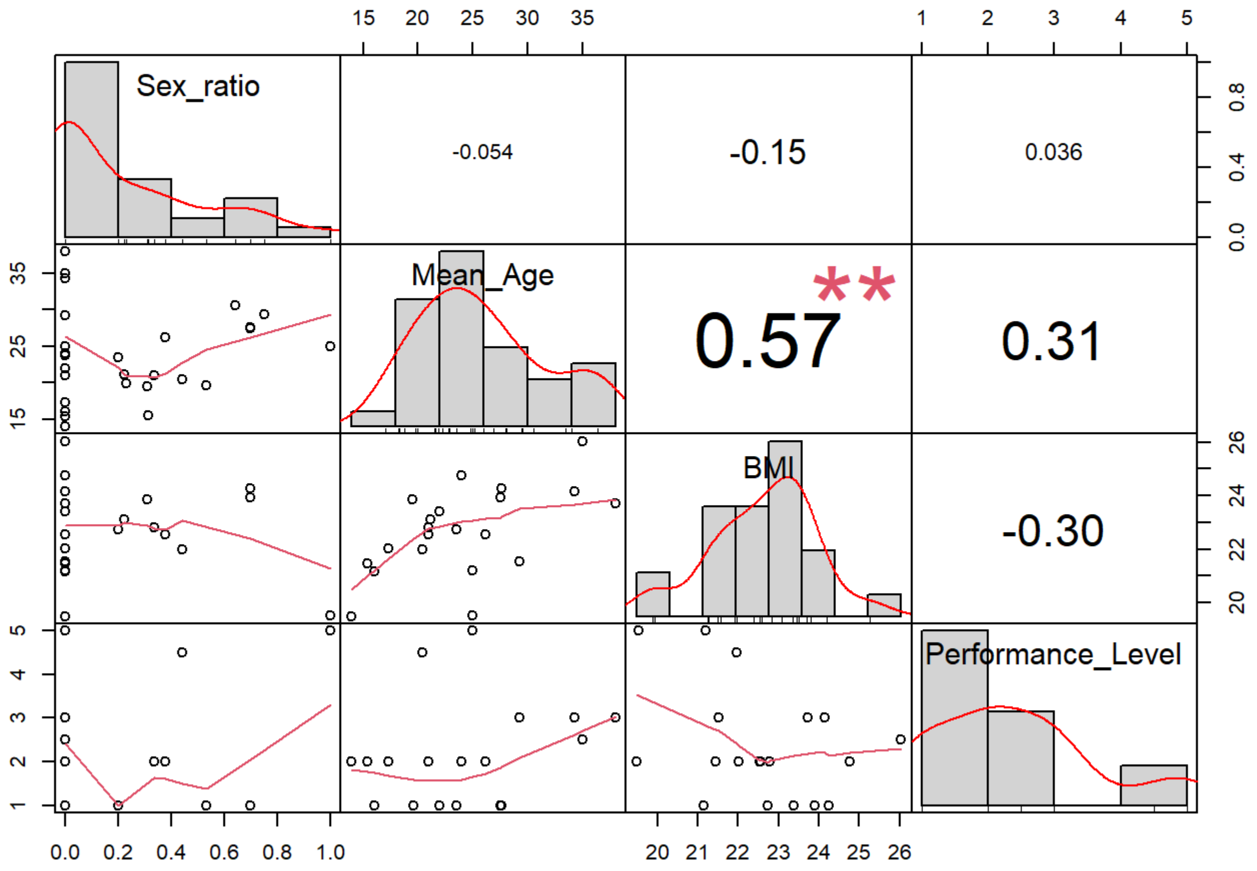

Fitting regression model

```
REMAMR <- rma(yi = Hedges_g,
              sei = SE_g,
              data = BasicMAfile,
              method = "ML",
              mods = ~ Sex_ratio + Mean_Age + BMI + Performance_Level,
              test = "knha")

REMAMR
```

```
##
## Mixed-Effects Model (k = 22; tau^2 estimator: ML)
##
## tau^2 (estimated amount of residual heterogeneity):     0.0582 (SE = 0.0615)
## tau (square root of estimated tau^2 value):             0.2413
## I^2 (residual heterogeneity / unaccounted variability): 27.66%
## H^2 (unaccounted variability / sampling variability):   1.38
## R^2 (amount of heterogeneity accounted for):            18.97%
##
## Test for Residual Heterogeneity:
## QE(df = 17) = 26.8136, p-val = 0.0609
##
## Test of Moderators (coefficients 2:5):
## F(df1 = 4, df2 = 17) = 0.6124, p-val = 0.6594
##
## Model Results:
##
##                    estimate      se     tval   df     pval     ci.lb    ci.ub    <U+200B>
```

```
## intrcpt             -1.5404  2.0242  -0.7610  17  0.4571  -5.8111  2.7303
## Sex_ratio            0.5322  0.3484   1.5276  17  0.1450  -0.2028  1.2672
## Mean_Age            -0.0031  0.0207  -0.1509  17  0.8819  -0.0467  0.0405
## BMI                  0.0493  0.0984   0.5006  17  0.6231  -0.1583  0.2568
## Performance_Level   -0.0058  0.0996  -0.0583  17  0.9542  -0.2160  0.2044
##
## ---
## Signif. codes:  0 '***' 0.001 '**' 0.01 '*' 0.05 '.' 0.1 ' ' 1
```
