# Supplementary material for: Interindividual Variability in Mental Fatigue-Related Impairments in Endurance Performance: A Systematic Review and Multiple Meta-regression
Source: Sports Med Open. 2023 Feb 20;9:14. doi: 10.1186/s40798-023-00559-7 (PMC9941412; doi:10.1186/s40798-023-00559-7)
Supplement: Supplementary file 3 — Additional file 3. Meta regression result. [file 40798_2023_559_MOESM3_ESM.pdf]

Supplementary Electronic Material 3: Meta Regression

Article title:

Interindividual Variability in Mental Fatigue-Related Impairments in Endurance Performance: a Systematic Review and Multiple Meta-Regression

Journal name:

Sports Medicine Open

Author names:

Jelle Habay[1,2,3,4], Robin Uylenbroeck[1], Ruben Van Droogenbroeck[1], Jonas De Wachter[1,2], Matthias Proost[1,2], Bruno Tassignon[1,2], Kevin De Pauw[1,2], Romain Meeusen[1,2], Nathalie Pattyn[1,3], Jeroen Van Cutsem[1,3], Bart Roelands[1,2]

Affiliation:

[1] Human Physiology and Sports Physiotherapy Research Group, Faculty of Physical Education and Physiotherapy, Vrije Universiteit Brussel, Belgium.

[2] BruBotics, Vrije Universiteit Brussel, Brussels, Belgium

[3] Vital Signs and Performance Monitoring Research Unit, LIFE Department, Royal Military Academy, Brussels, Belgium

[4] Research Foundation Flanders (FWO), Brussels, Belgium

Corresponding author:

Prof. Dr. Bart Roelands; Faculty of Physical Education and Physiotherapy, Human Physiology and Sports Physiotherapy Research Group, Vrije Universiteit Brussel, Pleinlaan 2, 1050, Brussels, Belgium; bart.roelands@vub.be; 0032 2 629 28 75

Supplementary electronic material C. Results of the Multiple Meta Regression Model

Factor	Represented by	Estimate	SE	t	p	95% CI
Sex (x_1)	Sex ratio (amount of women/total sample size)	0.53	0.35	1.53	0.1450	[-0.20; 1.27]
Age (x_2)	Mean age (y)	-0.003	0.02	-0.15	0.8819	[-0.05; 0.04]
BMI (x_3)	Weight (kg)/ (Height (cm))^2	0.05	0.09	0.47	0.6437	[-0.14; 0.23]
Performance Level (x_4)	Values determined by De Pauw et al. [1] and Decroix et al. [2]	0.006	0.10	-0.06	0.9542	[-0.22; 0.20]
Intercept		-1.54	2.02	-0.76	0.4571	[-5.81; 2.73]

Regression equation

$$y \text{ (Hedges g)} = 0.53x_1 - 0.003x_2 + 0.05x_3 + 0.006x_4 - 1.54$$

Mixed effect model based on k = 22 effects:

- tau^2 (estimated amount of residual heterogeneity): 0.0582 (SE = 0.0615)
- tau (square root of estimated tau^2 value): 0.2413
- I^2 (residual heterogeneity / unaccounted variability): 27.66%
- H^2 (unaccounted variability / sampling variability): 1.38
- R^2 (amount of heterogeneity accounted for): 18.97%

Test for Residual Heterogeneity:

QE(df = 17) = 26.8136, p = 0.0609

Test of Moderators (coefficients 2:5):

F(df1 = 4, df2 = 17) = 0.6124, p = 0.6594

CI = confidence interval; cm = centimetres; kg = kilograms; SE = standard error; (y) = years)

1. De Pauw K, Roelands B, Cheung SS, De Geus B, Rietjens G, Meeusen R. Guidelines to classify subject groups in sport-science research. Human Kinetics Publishers Inc.; 2013. p. 111-22.

Supplementary electronic material C. Results of the Multiple Meta Regression Model

2. Decroix L, De Pauw K, Foster C, Meeusen R. Guidelines to Classify Female Subject Groups in Sport-Science Research. Int J Sports Physiol Perform. 2016 Mar;11(2):204-13.
